# Roquin recognizes a non-canonical hexaloop structure in the 3′-UTR of *Ox40*

**DOI:** 10.1038/ncomms11032

**Published:** 2016-03-24

**Authors:** Robert Janowski, Gitta A. Heinz, Andreas Schlundt, Nina Wommelsdorf, Sven Brenner, Andreas R. Gruber, Michael Blank, Thorsten Buch, Raymund Buhmann, Mihaela Zavolan, Dierk Niessing, Vigo Heissmeyer, Michael Sattler

**Affiliations:** 1 Group Intracellular Transport and RNA Biology, Institute of Structural Biology, Helmholtz Zentrum München, Ingolstädter Landstrasse 1, Neuherberg DE-85764, Germany; 2 Research Unit Molecular Immune Regulation, Helmholtz Zentrum München, Marchioninistrasse 25, München DE-81377, Germany; 3 Institute for Immunology at the Biomedical Center, Ludwig-Maximilians-Universität München, Grosshaderner Strasse 9, Planegg-Martinsried DE-82152, Germany; 4 Institute of Structural Biology, Helmholtz Zentrum München, Ingolstädter Landstrasse 1, Neuherberg DE-85764, Germany; 5 Center for Integrated Protein Science Munich at Biomolecular NMR Spectroscopy, Department Chemie, Technische Universität München, Lichtenbergstrasse 4, Garching DE-85747, Germany; 6 Biozentrum, University of Basel and Swiss Institute of Bioinformatics, Klingelbergstrasse 50–70, Basel CH–4056, Switzerland; 7 AptaIT GmbH, Goethestrasse 52, München 80336, Germany; 8 Institute of Laboratory Animal Science, University of Zurich, Wagistrasse 12, Schlieren 8952, Switzerland; 9 Department of Medicine III and Transfusion Medicine, University Hospital Grosshadern, LMU, Marchioninistrasse 15, München 81377, Germany; 10 Department of Cell Biology at the Biomedical Center, Ludwig-Maximilians-Universität München, Grosshaderner Strasse 9, Planegg-Martinsried DE-82152, Germany

## Abstract

The RNA-binding protein Roquin is required to prevent autoimmunity. Roquin controls T-helper cell activation and differentiation by limiting the induced expression of costimulatory receptors such as tumor necrosis factor receptor superfamily 4 (Tnfrs4 or Ox40). A constitutive decay element (CDE) with a characteristic triloop hairpin was previously shown to be recognized by Roquin. Here we use SELEX assays to identify a novel U-rich hexaloop motif, representing an alternative decay element (ADE). Crystal structures and NMR data show that the Roquin-1 ROQ domain recognizes hexaloops in the SELEX-derived ADE and in an ADE-like variant present in the *Ox40* 3′-UTR with identical binding modes. In cells, ADE-like and CDE-like motifs cooperate in the repression of *Ox40* by Roquin. Our data reveal an unexpected recognition of hexaloop *cis* elements for the posttranscriptional regulation of target messenger RNAs by Roquin.

## Abstract (web summary)

Roquin is an RNA-binding protein that prevents autoimmunity by limiting expression of receptors such as Ox40. Here, the authors identify an RNA structure that they describe as an alternative decay element, and they characterise its interaction with Roquin using structural and biochemical techniques.

## Graphical abstract

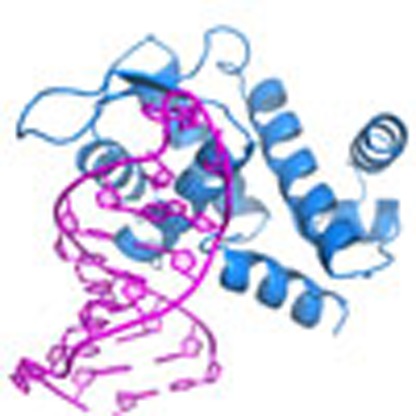

Posttranscriptional gene regulation is involved in a wide range of cellular functions and its critical importance has been described for many developmental and differentiation processes^1^. Consistently, mutations of factors involved in posttranscriptional gene regulation pathways were found associated with a number of genetically inherited diseases^2^. The Roquin protein is essential in T cells for the prevention of autoimmune disease. This is evident from the so-called *sanroque* mutation in Roquin-1, a single amino acid exchange from Met199 to Arg that causes the development of systemic lupus erythematosus-like symptoms in homozygous mice^3^. The *Rc3h1* and *Rc3h2* genes, encoding for Roquin-1 and Roquin-2 proteins in vertebrates, respectively, have both been shown to be essential for the survival of mice, but apparently serve redundant functions in T cells^4^,^5^,^6^,^7^. Consistently, CD4^+^ and CD8^+^ T cells with the combined deletion of Roquin-encoding genes are spontaneously activated and CD4^+^ T-helper cells preferentially differentiate into the Th1, Tfh or Th17 subsets^7^,^8^. Roquin-1 was shown to negatively regulate expression of transcripts encoding for co-stimulatory receptors such as Icos, Ox40 and CTLA-4, for cytokines such as interleukin (IL)-6 and tumour necrosis factor or for transcription factors such as IRF4, IκBNS and IκB*ζ* (refs 7, 8, 9, 10).

We have recently reported structural and functional data of the Roquin-1 ROQ domain bound to a canonical constitutive decay element (CDE), a short stem loop (SL) that acts as a *cis*-regulatory RNA element in the 3′-untranslated regions (3′-UTRs) of target genes such as *Tnf* (ref 11). The ROQ domain adopts an extended winged helix fold that engages predominantly non-sequence-specific protein–RNA contacts and mainly recognizes the shape of the canonical *Tnf* CDE RNA. The structural data and mutational analysis indicated that a broader, extended range of sequence variations in both the loop and stem of the CDE element is recognized and regulated by Roquin. At the same time, Tan *et al*.^12^ described the crystal structure and supporting functional data of a similar interaction with a CDE-like SL, and reported a second binding site for a double-stranded RNA (dsRNA) within an extended ROQ domain. The structural basis for CDE recognition by the Roquin-2 ROQ domain has also been recently reported^13^.

We found that the posttranscriptional activity of Roquin-1 and Roquin-2 is regulated through cleavage by the paracaspase MALT1 (refs 8, 14). Enhanced MALT1-dependent cleavage and inactivation of Roquin, and thus less effective repression of target genes, result from increased strength of antigen recognition in T cells^8^. These findings suggest that dependent on the strength of cognate antigen recognition differential gene expression and cell fate decisions can be established in naive T cells by a graded cleavage and inactivation of Roquin. In addition to this mechanism, the composition and binding affinity of *cis*-regulatory SL elements in the 3′-UTRs of target mRNAs may determine the sensitivity to repression by the *trans*-acting factor Roquin. Defining the SL RNA structures that are recognized by Roquin is therefore essential for our understanding of posttranscriptional gene regulation by Roquin and its involvement in T-cell biology and T-cell-driven pathology.

Here we present structural and functional evidence for a greatly expanded repertoire of RNA elements that are regulated by Roquin as demonstrated with a novel U-rich hexaloop SL in the 3′-UTR of *Ox40* bound to the Roquin-1 ROQ domain. We find an additive regulation of *Ox40* gene expression based on both its CDE-like and hexaloop SL RNAs that we identified using Systematic Evolution of Ligands by Exponential Enrichment (SELEX) experiments. Our X-ray crystallographic, NMR, biochemical and functional data combined with mutational analysis demonstrate that both triloop and hexaloop SL RNAs contribute to the functional activity of Roquin in T cells.

## Results

### SELEX identifies novel RNA ligands of Roquin-1

**Figure 1:**
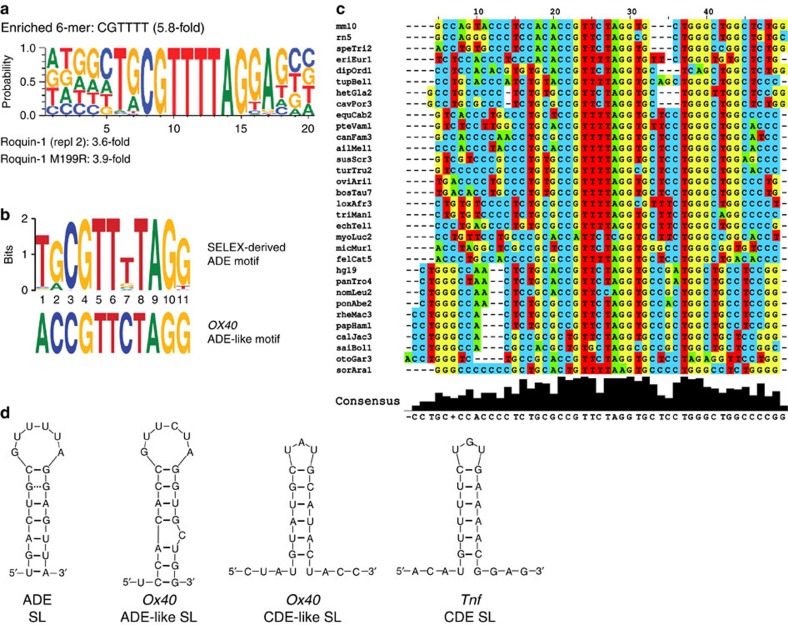
SELEX identifies a novel SL RNA ligand of Roquin-1. (**a**) Enriched hexamers that were found by Roquin-1 N terminus (residues 2–440) or Roquin-1 M199R N terminus (residues 2–440) (see also Supplementary Fig. 1). (**b**) An ADE sequence motif in the *Ox40* 3′-UTR closely resembles the MEME motif found in SELEX-enriched RNA sequences. (**c**) Conservation of the motif found in *Ox40* 3′-UTRs for various species as indicated. The labels correspond to the versions of the genome assemblies in the UCSC server (see Method section). rn5 is the fifth assembly version of the rat (*Rattus novegicus*). (**d**) Schematic representation of the predicted SELEX-derived consensus SL, ADE and the *Ox40* ADE-like hexaloop SL. The broken line between the G–G base pair in the ADE SL indicates a putative non-Watson–Crick pairing. The *Ox40* CDE-like SL and the *Tnf* CDE SL are shown for comparison. See also Supplementary Fig. 1.

We set out to identify Roquin-bound RNA motifs in an unbiased manner by performing SELEX experiments. A biotinylated amino-terminal protein fragment of Roquin-1 (residues 2–440) was used to enrich RNAs from a library containing 47 random nucleotides over three sequential selection rounds. Next-generation sequencing (NGS) of the RNA before and after each selection round revealed that the starting pool represented about 99.6% unique reads in ∼4.2 × 10^6^ sequences. Bioinformatic analysis of NGS data sets derived from the starting pool and enriched selection rounds revealed that the complexity was reduced to 78.6% unique reads in 3.7 × 10^6^ sequences that were analysed after 3 rounds of selection and enrichment. For NGS data analysis, the COMPAS software (AptaIT, Munich, Germany) was applied. Enriched sequences were clustered into so-called patterns with highly homologous sequences. Hereby, the algorithm at first identified frequent motifs of five to eight nucleotides length and subsequently used iterative cycles of proto-pattern formation to cluster sequences bearing two of such frequent motifs. A final aptamer pattern was built up by sequences bearing two frequent motifs and, at the same time, having high similarities also in other sequence parts. Based on this so-called co-occurrence approach, patterns on the basis of frequent motifs were generated and were searched for prominent hexamer sequences (Supplementary Fig. 1a). We identified 5′-CGTTTT-3′, 5′-GCGTTT-3′, 5′-TGCGTT-3′ and 5′-GTTTTA-3′ motifs that were also reconfirmed in an independent experiment (Supplementary Fig. 1a) and are located within highly similar sequences (Fig. 1a and Supplementary Fig. 1b). Consistent with previous findings showing that the *sanroque* mutation does not impair RNA binding of Roquin^15^, we found similarly enriched sequences in SELEX approaches using a corresponding Roquin-1 fragment harbouring the M199R mutation (Fig. 1a and Supplementary Fig. 1b). Notably, our SELEX approach did not reveal the previously identified CDE sequence. We assume that the region of sequence identity in the CDE is too short for our sequence clustering algorithm. Evaluation of the structural context for the SELEX-derived motif suggested a putative SL formation with six unpaired nucleotides in a loop followed by a 5–8 nt stem, with one base in the stem not being paired (Supplementary Fig. 1c). Searching the 3′-UTRs of known Roquin targets with the consensus 5′-TGCGTTTTAGGA-3′, obtained by Motif-based sequence analysis (MEME), revealed a homologous sequence with the potential to form a hexaloop structure in the 3′-UTR of *Ox40* (Fig. 1b). Importantly, this motif is present across species in the 3′-UTRs of respective mRNAs and showed highest conservation in the loop and the upper stem sequences with a drop of conservation towards the boundaries of the motif (Fig. 1c,d). The predicted SL for the consensus SELEX-derived motif (from here on referred to as alternative decay element SL, ADE SL), the ADE-like SL, is positioned 5′ to another CDE-like SL in the 3′-UTR of *Ox40* mRNA^11^. This CDE-like SL differs in the sequence of the upper stem from the canonical CDE from the 3′-UTR of *Tnf* mRNA (CDE SL) (Fig. 1d).

### NMR analysis of Roquin-bound SL RNAs

**Figure 2:**
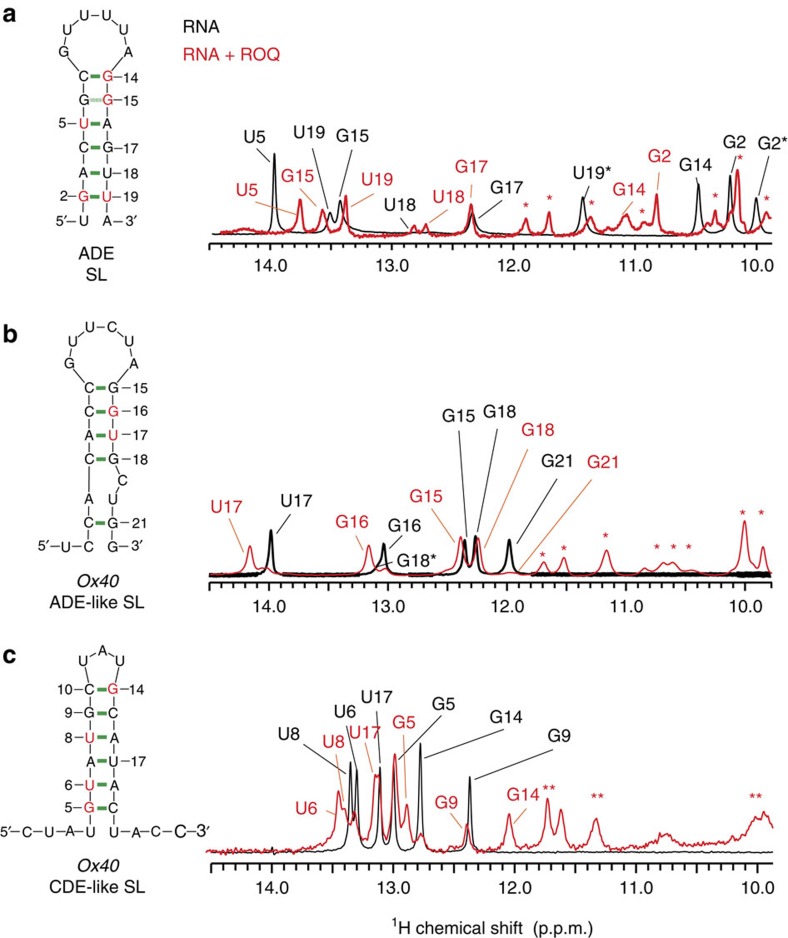
NMR analysis of the SL RNAs used in this study. Imino proton regions of one-dimensional ^1^H NMR spectra of (**a**) the ADE SL (**b**), the *Ox40* ADE-like SL and (**c**) the *Ox4*0 CDE-like SL are shown for free RNAs (black) and in complex with the Roquin-1 ROQ domain (red). The respective SL RNAs and their base pairs are indicated. Red asterisks indicate NMR signals of the protein. Black asterisks in **a** indicate a second conformation (see Supplementary Notes). Green lines in the secondary structure schemes on the left refer to visible imino NMR signals and thus experimental confirmation of the base pairs indicated. Red nucleotides indicate significant chemical shift changes observed. The dotted green line between G6 and G15 in **a** highlights a G–G base pair.

We used NMR to analyse the secondary structure of Roquin-1-binding motifs derived from SELEX. Imino one- and two-dimensional nuclear Overhauser enhancement spectroscopy (NOESY) NMR spectra of the free RNA and when bound to the Roquin-1 ROQ domain were recorded for the ADE SL, the ADE-like SL in the 3′-UTR of *Ox40* and the previously identified *Ox40* CDE-like SL^11^ (Fig. 2). The NMR data of the free RNAs show that almost all predicted base pairs in the stem regions of the hexa- and triloop SL including the closing base pairs are formed in all three RNAs. Notably, we also found an unambiguous imino proton signal for G15, but not G6, in the ADE SL, indicating a non-Watson–Crick G–G base pair at this position (Fig. 2a). Significant chemical shift perturbations (CSPs) are observed for imino proton signals on binding to the ROQ domain, demonstrating that formation of protein–RNA complexes involves contacts of the ROQ domain to the stem region of the RNA ligands (Fig. 2, bases coloured red). No imino correlations are observed for the predicted Watson–Crick base pairs at the bottom of the ADE SL and the *Ox40* ADE-like SL RNAs, as well as for the A–U base pair flanking the bulge in the *Ox40* ADE-like SL RNA (Fig. 2a,b), suggesting that these base pairs are dynamic. In contrast, all expected base pairs are observed for the *Ox40* CDE-like SL RNA (Fig. 2c; see also Supplementary Notes).

### Structures of ROQ bound to ADE SL RNAs

**Figure 3:**
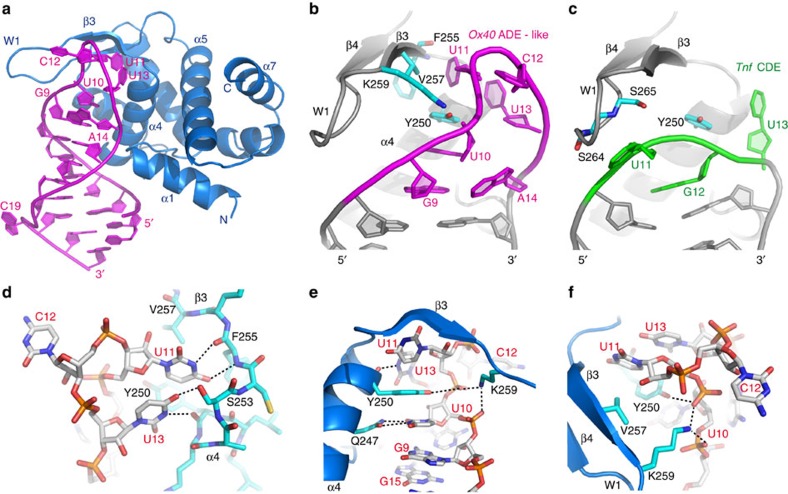
Structure of the Roquin-1 ROQ domain bound to *Ox40* ADE-like RNA. (**a**) Cartoon presentation of the crystal structure of the ROQ domain (residues 174–325; blue) and the *Ox40* ADE-like SL RNA (magenta). Selected RNA bases and protein secondary structure elements are labelled. (**b**) Close-up view of the *Ox40* ADE-like SL (bases in the RNA hexaloop are shown in magenta) and (**c**) the previously reported structure of the ROQ-*Tnf* CDE complex^11^ (bases of the triloop RNA are shown in green). Only RNA-interacting residues that are different in both structures are shown. Both protein chains and remaining parts of both RNAs are shown in grey and protein residue side chains are shown in turquoise. (**d**) Close-up view of the contacts between the ROQ domain and nucleotides U11 and U13 of the *Ox40* ADE-like SL RNA. The nucleotides interact with the C-terminal end of helix α4 (Tyr250 and Ser253) and the N-terminal part of strand β3 (Phe255 and Val257). The protein chain is shown in turquoise and the RNA is shown in grey. Atoms are colour coded according to charge. (**e**) Close-up view of the contacts between the ROQ domain and nucleotides U10, U11 and U13 in the RNA hexaloop. U11 and U13 contact the C-terminal end of helix α4: residues Tyr250 and Gln247. The side chain of Tyr250 makes hydrophobic interactions with the pyrimidine side chain of U10 on one side and U11 on the other side. Lys259 interacts with the phosphate groups of U10 and U11. (**f**) Close-up view of the hydrophobic interaction between Val257 and U11, as well as the double hydrogen bond of Lys259 with phosphate groups of U10 and U11. In **d** – **f**, amino acids are shown in turquoise and blue, nucleotides in grey colour. See also Supplementary Notes and Supplementary Fig. 2.

To elucidate how Roquin can recognize the novel SL elements identified in the SELEX approach, we solved crystal structures of the Roquin-1 ROQ domain bound to these non-canonical RNA elements. The structures of ROQ bound to the 20-mer ADE SL (Supplementary Fig. 2a) and to the 22-mer *Ox40* ADE-like SL RNAs (Fig. 3a) were refined to a resolution of 3.0 and 2.2 Å, respectively. In both structures the RNA adopts an SL fold, where the hexaloop is located in the vicinity of the carboxy-terminal end of ROQ helix α4 and the N-terminal part of β3 (Fig. 3a,b and Supplementary Fig. 2a,b). The dsRNA stem is recognized in the same way as previously reported for the *Tnf* CDE SL RNA (Supplementary Fig. 2c–e)^11^. As may be expected, the recognition of the hexaloop is significantly different from the triloop in the CDE RNA (Fig. 3b,c and Supplementary Fig. 2b). Interestingly, although the sequences of the ADE SL and ADE-like SL RNAs are different, the overall structures and protein–RNA contacts are virtually identical (Supplementary Fig. 2a,d,e). The only differences are a C19 bulge, the non-Watson–Crick G6–G15 base pair and the interaction of U1 with Trp184 and Phe194 in the ADE-like SL RNA (Supplementary Fig. 2a,e–g). Given their highly similar binding modes we focus the following discussion on the structure of the *Ox40* ADE-like SL RNA, as it naturally exists in the *Ox40* 3′-UTR and was solved at higher resolution.

The overall orientation and recognition of the double-stranded stem in the *Ox40* ADE-like SL is similar to the CDE triloop^11^. Notably, the U-rich hexaloop in the *Ox40* ADE-like SL RNA binds to an extended surface on the ROQ domain that cannot be accessed by the CDE triloop^11^ (Fig. 3b,c) and includes a few pyrimidine-specific contacts. For example, the main chain atoms of Phe255 form two hydrogen bonds with the Watson–Crick face of the U11 base (Fig. 3d). Although in the structure of the *Tnf* CDE triloop the Tyr250 side chain engages only one hydrogen bond to the phosphate group of G12 (ref. 11), a number of contacts are observed with the hexaloop (Fig. 3d–f): the side chain hydroxyl of Tyr250 contacts the phosphate group of U11, while the aromatic ring is positioned by parallel and orthogonal stacking interactions with the U10 and U11 bases, on either side, respectively (Fig. 3e). In addition, the Tyr250 main-chain carbonyl interacts with U13 imino proton (Fig. 3d,e). Val257 and Lys259 in strand β3 are too far to contact the UGU triloop in the *Tnf* CDE RNA^11^, but mediate a number of contacts with the longer hexaloop. The side chain of Lys259 forms hydrogen bonds with the phosphate groups of U10 and U11 (Fig. 3e,f) and the hydrophobic side chain of Val257 stacks with the U11 base (Fig. 3d,f). The RNA stem is closed by a Watson–Crick base pair (C8–G15 in the hexaloop SL RNA). Interestingly, the G9 base stacks on top of this closing base pair and takes a position that is very similar to the purine base of G12 in the CDE triloop (Fig. 3b,c and Supplementary Fig. 2b). The G9 base does not form a base pair with A14 but rather the A14 base packs into the minor groove of the RNA duplex. This arrangement provides an extended stacking interaction of G9, U10 and Tyr250 in the ROQ domain at the 5′-side of the RNA stem (Fig. 3e). The U11 and U13 bases stack with each other in the vicinity of the ROQ domain wing (Fig. 3b,d,f). This is possible by exposing the base C12 of the *Ox-40* ADE-like SL towards the solvent, which accordingly does not show any contacts to the protein. In summary, similar to the CDE SL, both the ADE SL and ADE-like SL RNAs are recognized mainly by non-sequence-specific contacts. However, these involve an extended binding surface on the ROQ domain with a number of additional residues compared with the triloop RNA.

### NMR analysis of ROQ interactions with ADE SLs

**Figure 4:**
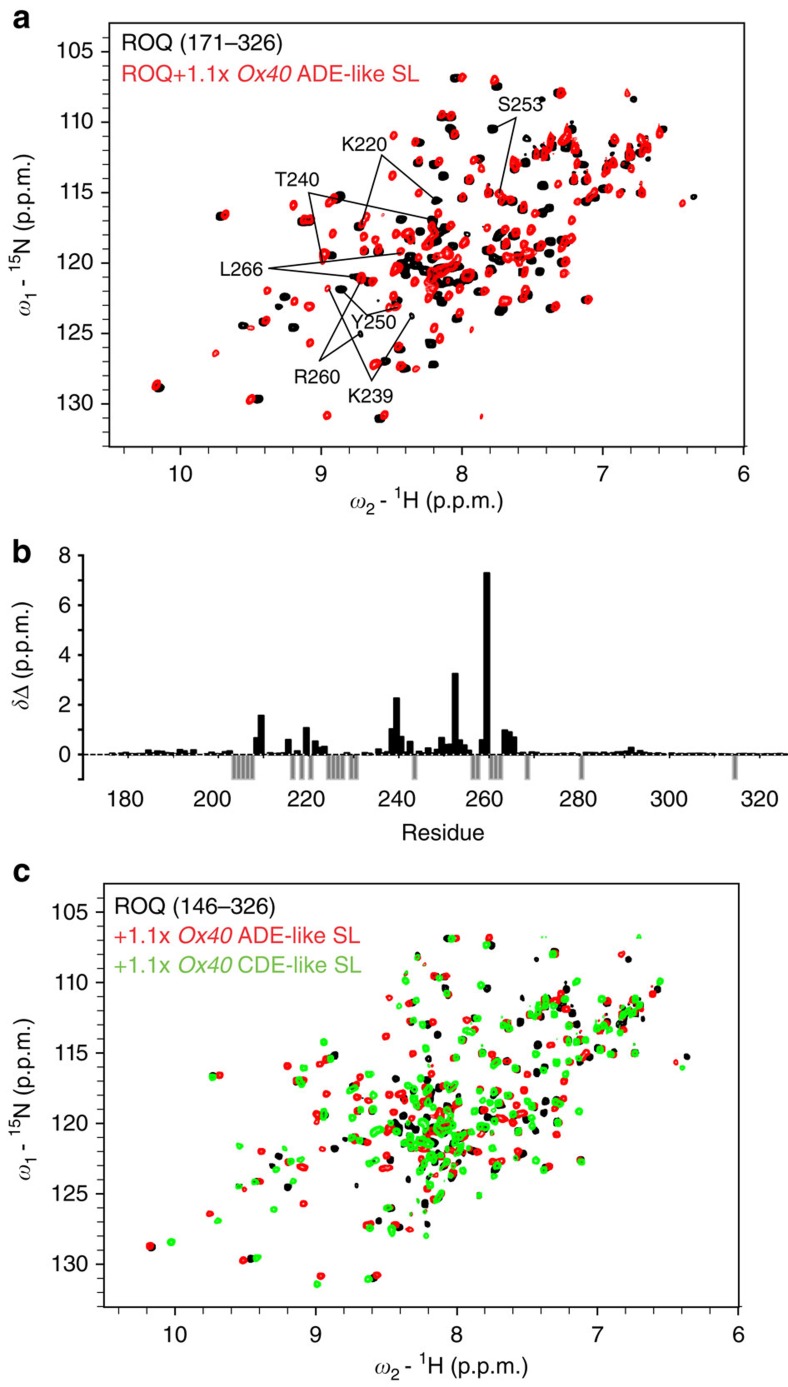
NMR analysis of ROQ domain interactions with the *Ox40* ADE-like hexaloop RNA. (**a**) Overlay of ^1^H,^15^N HSQC spectra of either the free ROQ domain (171–326, black) or in complex with stoichiometric amounts of the *Ox40* ADE-like SL (red). Selected shifts of amide resonances are indicated. (**b**) Plot of chemical shift change versus residue number in the ROQ domain (residues 171–326) from **a**. Grey negative bars indicate missing assignments in one of the spectra. Gaps indicate prolines. (**c**) Overlay of the ROQ domain alone (black) or in complex with the *Ox40* ADE-like SL (red) or the *Ox40* CDE-like SL (green). See also Supplementary Notes and Supplementary Fig. 3.

We next used NMR spectroscopy to compare the ROQ domain interaction of ADE-like and CDE-like SL RNAs in solution. CSPs observed for amides in the ROQ domain on binding to the *Ox40* ADE-like SL RNA (Fig. 4a,b) map to residues that also mediate key interactions with CDE SLs^11^, such as Lys220, Lys239/Thr240 and Lys259/Arg260 (Fig. 4b). This is fully consistent with the interactions observed in the crystal structure (Supplementary Fig. 2c–e) and indicates a similar binding surface. However, there are also notable CSP differences when comparing binding of the ROQ domain to *Ox40* ADE-like SL RNAs and to the CDE-like SL RNA in the *Ox40* 3′-UTR (Fig. 4c), or to the *Tnf* CDE SL RNA (Supplementary Fig. 3 and Supplementary Notes). For example, Ser253 is strongly affected only on binding to the *Ox40* ADE-like SL (Fig. 4a,b) in line with tight interactions with the hexaloop (Fig. 3d). On the other hand, comparison of ROQ domain binding with the ADE and with the ADE-like SL RNAs indicates almost identical NMR spectra and CSPs. This is consistent with the very similar structural features and mode of RNA recognition of the ROQ domain with these RNAs (Supplementary Fig. 2a,d,e).

### Mutational analysis of the ROQ-ADE interaction

**Figure 5:**
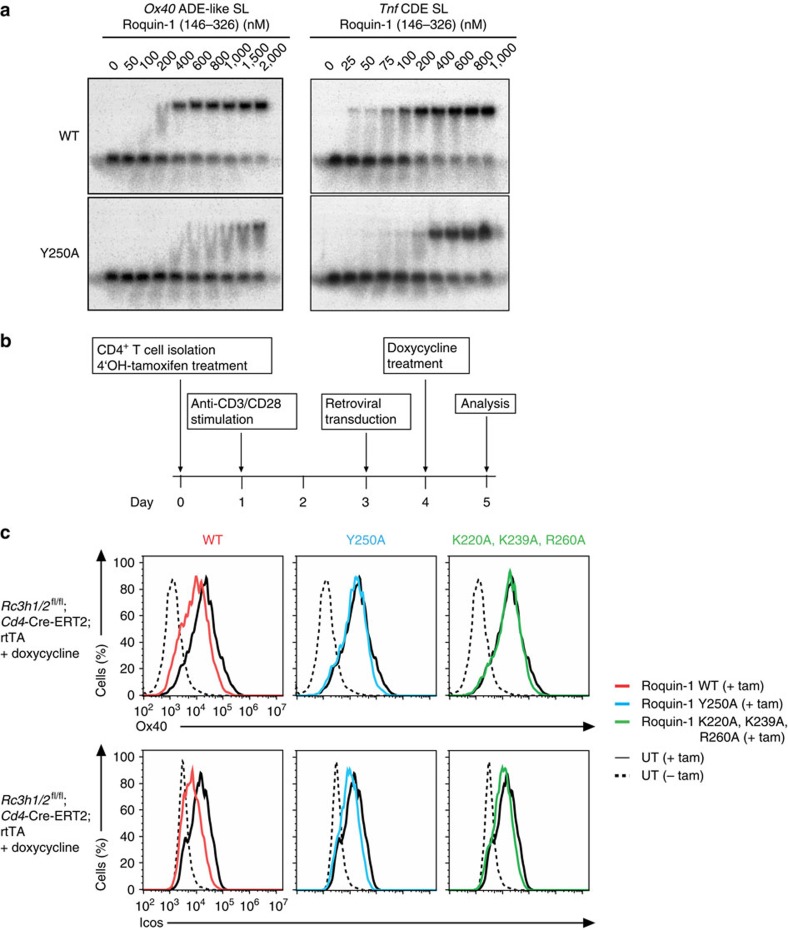
Mutational analysis of Roquin-1-interactions with *Ox40* ADE-like SL and *Ox40* 3′-UTR. (**a**) EMSA assay comparing binding of the wild-type and of the Y250A mutant ROQ domain for binding to the *Ox40* ADE-like SL (left) or the previously described *Tnf* CDE SL (right). A comparison of further mutants is shown in Supplementary Fig. 4. (**b**) Schematic overview of the timeline used for the reconstitution experiment shown in **c**. (**c**) Flow cytometry of Ox40 and Icos surface expression on CD4^+^ T_h_1 cells from *Rc3h1/2*^fl/fl^; *Cd4*-Cre-ERT2; rtTA mice treated with tamoxifen (+tam) to induce Rc3h1/2^fl/fl^ deletion or left untreated (− tam). The cells were then either left untransduced (UT) or were transduced with retrovirus containing a doxycycline-inducible cassette, to express Roquin-1 WT, Roquin-1 Y250A or Roquin-1 K220A, K239A and R260A mutants (see also Supplementary Fig. 5).

To examine the individual contributions of ROQ–hexaloop interactions for complex formation, we performed electrophoretic mobility shift assays (EMSAs) with variants of the ROQ domain and the *Ox40* ADE-like RNA (Fig. 5a and Supplementary Fig. 4). Analysis of the interaction with wild-type ROQ revealed an apparent affinity in a similar range as for the *Tnf* CDE^9^,^11^ (Fig. 5a and [Table t1]) Table 2). We next tested a set of mutants (Supplementary Fig. 4), which were designed based on contacts observed in the crystal structure (Fig. 3) and the NMR CSPs (Fig. 4a,b). In line with expectations from ROQ-*Tnf* CDE binding (see comparison in Supplementary Fig. 4) and based on our structural analysis, the key residues Lys220, Lys239, Lys259 and Arg260 strongly reduce or abolish binding after replacement by alanine. We also observe an almost complete loss of binding in the Y250A mutant to the hexaloop SL RNA, which had not been seen for the *Tnf* CDE previously^11^ (Fig. 5a). This underlines the central role of Tyr250 for stabilization of the hexaloop structure and recognition by stacking interactions (Fig. 3b,e). Mutation of Ser253, which shows large CSPs in the NMR titrations (Fig. 4a,b), does not significantly impair complex formation (Supplementary Fig. 4). The large chemical shift change is probably caused by ring current effects induced by the close proximity of the U11 and U13 bases. Finally, a mutant in the wing of the ROQ domain (S265Y) does only slightly impair binding, as has been previously observed for the interaction with the *Tnf* CDE^11^ (Supplementary Fig. 4). This indicates that replacement by Tyr does not strongly affect the RNA interaction, and that some conformational variations are tolerated. Thus, the mutational analysis is fully consistent with the recognition of the hexaloop observed in our crystal structures.

To prove the contribution of the key residue Tyr250 in Roquin-1 to *Ox40* mRNA recognition and regulation, we set up a retroviral reconstitution system in Roquin-deficient CD4^+^ T cells. Isolated CD4^+^ T cells from *Rc3h1/2*^fl/fl^; *Cd4*-Cre-ERT2; rtTA mice harbouring floxed Roquin-1/2 encoding alleles, a tamoxifen-inducible Cre recombinase and the reverse tetracycline-controlled transactivator rtTA were treated *in vitro* with 4-hydroxy tamoxifen, to induce deletion. The cells were then transduced with doxycycline-inducible retroviral vectors to reconstitute Roquin-1 expression (Fig. 5b). Depletion of Roquin proteins on tamoxifen treatment (Supplementary Fig. 5a) strongly increased surface expression of Ox40 and Icos (Fig. 5c). This increase in surface expression of both costimulatory receptors was partially corrected by the doxycycline-induced reconstitution with Roquin-1 WT protein (Fig. 5c left panels). Importantly, no effect was observed on expression of the Y250A mutant of Roquin-1 or the K220A, K239A and R260 mutant, which is strongly impaired in CDE SL interactions (Fig. 5c middle and right panels). The observed partial rescue may relate to the low, close to endogenous expression of these constructs (Supplementary Fig. 5b). However, it is also possible that continuous overexpression of targets following Roquin deletion induces a hyperactivated state in the T cells. This hyperactivation, compared with the actual posttranscriptional derepression, may contribute even stronger to the increased Icos and Ox40 expression levels. Hence, our structure–function analyses conclusively show that the Y250 residue is essential for Roquin interaction and regulation of *Ox40*, and potentially also for other Roquin targets such as *Icos*.

We also investigated the role of individual nucleotides in the *Ox40* ADE-like SL for complex formation with the ROQ domain. We designed four mutants (Mut1–4, see Supplementary Fig. 6) that were expected to disrupt key interactions with the protein according to our co-crystal structure (Fig. 3d–f and Supplementary Fig. 2). NMR analysis confirmed that all mutant RNAs formed the same base pairs in the stem region, identical to the wild-type ADE-like SL (Fig. 2b and Supplementary Fig. 6). We next used surface plasmon resonance experiments to determine dissociation constants for the ROQ-RNA interaction (Table 2 and Supplementary Fig. 7). Although the replacement of a C8–G15 closing base pair by A-U (Mut 4) only reduces the affinity threefold, reduction of loop size in the A14C mutant (Mut 1, see Table 2) reduces the affinity and binding is not detected by surface plasmon resonance. As intended, the mutation Mut 1 allows the formation of an additional base pair and thus leads to the formation of a tetraloop with a new G-C closing base pair (Supplementary Fig. 6a). Consistent with the structural analysis, we assume that this variant alters the hexaloop conformation and thus reduces the interaction with ROQ. Disruption of stacking interactions between G15, G9 and Y250 in the G9C mutant (Mut 2) completely abolished binding of ROQ to the SL RNA (Table 2 and Supplementary Fig. 7). No binding is also observed for the U11AU13G double mutant (Mut 3) (Table 2 and Supplementary Fig. 7), which abolishes specific interactions mediated by U11 and U13 in the hexaloop with ROQ (Fig. 3d). Consistent with the SELEX consensus (Fig. 1b), all of the tested mutations of conserved nucleotides in the loop reduce or abolish the interaction with ROQ. Interestingly, the affinity of the wild-type *Tnf* CDE and the *Ox40* ADE-like SLs to ROQ are very similar (42 and 81 nM, respectively, Table 2 and Supplementary Fig. 7).

### Roquin binding to different SLs in the *Ox40* 3′-UTR

**Figure 6:**
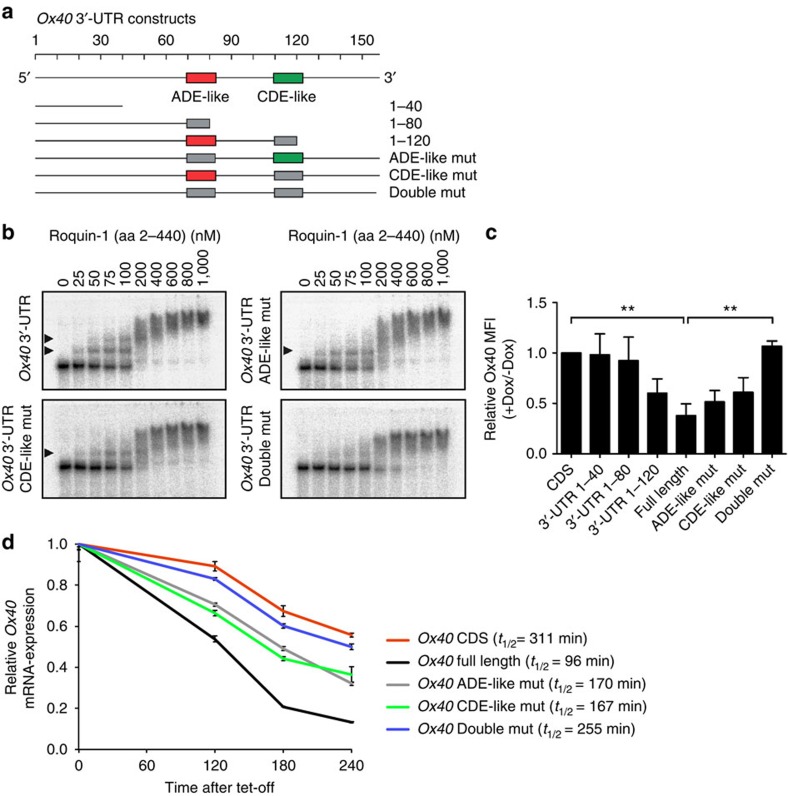
Functional importance of Roquin-1 target motifs in cells. (**a**) Overview of the *Ox40* 3′-UTR and truncated/mutated versions thereof as used for EMSA assays in **b** and the expression experiments of *Ox40* in **c** and **d**. (**b**) EMSA experiments probing the interaction between the Roquin-1 N-terminal region (residues 2–440) and either the complete wild-type *Ox40* 3′-UTR or versions with mutations of the CDE-like SL, the ADE-like SL or both SLs (see **a**). Arrows indicate the individual binding events to either motif. It is noteworthy that the higher bands observed at large protein concentrations are probably additional nonspecific, lower-affinity interactions of Roquin-1 with the 3′-UTR or protein aggregates. (**c**) Relative Ox40 MFI normalized to expression levels from the *Ox40* CDS construct. Error bars show s.d. of seven (CDS, 1–40, 1–80, 1–120 and full-length), six (ADE-like mut and CDE mut) or three (double mut) independent experiments. Statistical significance was calculated by one-way analysis of variance (ANOVA) Kruskal–Wallis test followed by Dunn’s multiple comparison test (**P<0.01). (**d**) mRNA decay curves of Hela Tet-Off cells stably transduced with retroviruses expressing *Ox40* CDS without 3′-UTR (CDS, red line), *Ox40* CDS with its wild-type 3′-UTR (full length, black line), *Ox40* full length with mutated ADE-like motif (ADE-like mut, grey line), *Ox40* full length with mutated CDE-like motif (CDE-like mut, green line) or *Ox40* full length with mutated ADE and CDE motifs (Double mut, blue line). Error bars represent the mean of technical duplicates in one experiment. mRNA half-life times were calculated with Graph Pad Prism. Data are representative of two experiments with similar results.

We have recently shown that Roquin-1 binds to a CDE-like motif in the 3′-UTR of *Ox40* mRNA^11^ (Figs 1d and 4c). We therefore investigated whether the interactions with the CDE-like and the ADE-like SL RNAs both contribute to Roquin-1 binding in the context of the full-length *Ox40* 3′-UTR. The binding affinities of either motif for the N-terminal domain of Roquin-1 (residues 2–440) (Supplementary Fig. 8a,b) or the ROQ domain alone are in a similar range (Table 2). The dissociation constants for the ROQ interaction with the *Ox40* CDE-like SL and the ADE-like SL RNAs are 1,460 and 81 nM, respectively (Table 2). This is consistent with the extended binding interface and additional interactions observed with the hexaloop, and suggests a preferential binding to the hexaloop SL RNA in the *Ox40* 3′-UTR. We designed different variants of the 3′-UTR by point mutagenesis abrogating base pairing in the stem region, where none, individual, or both SL RNA motifs were mutated to impair Roquin-1 binding (Fig. 6a). These RNAs were then tested in EMSAs with the Roquin-1 N terminus (residues 2–440) (Fig. 6b). Gel shift assays show that binding to the wild-type 3′-UTR construct leads to two distinct bands during the titrations, which should reflect binding to one and both RNA motifs, respectively. Consistent with this, both bands are strongly reduced when mutations are introduced that interfere with the formation of both SLs. Notably, among these, the slower migrating band disappears when either of the two SL RNA motifs is altered to impair Roquin binding, indicating an interaction with the remaining wild-type SL. We thus conclude that Roquin is able to bind to both SL RNA motifs in the context of the full-length *Ox40* 3′-UTR.

### Regulation of Ox40 expression via two motifs in its 3′-UTR

To investigate the role of the new ADE-like motif in target mRNA regulation, we introduced *Ox40* mRNA variants harbouring altered 3′-UTRs in cells. Considering the close proximity of the ADE-like and CDE-like SL RNAs in the 3′-UTR (Fig. 6a), which is essential for Roquin-mediated posttranscriptional regulation of Ox40 (ref. 7) we tested individual contributions and the functional cooperation of the two RNA elements by deletion and point mutagenesis abrogating base pairing in the stem region (Fig. 6a,c and Supplementary Fig. 8c). Specifically, using retroviruses we introduced *Ox40* expression constructs placed under the control of different 3′-UTRs into Roquin-1/2-deficient mouse embryonic fibroblasts. Doxycycline treatment of cells from this cell line enabled ectopic Roquin-1 and co-translational mCherry expression due to the stable integration of an inducible lentiviral vector (Supplementary Fig. 8c)^8^,^16^. The expression of Ox40 in cells with and without doxycycline treatment was then quantified by flow cytometry (Supplementary Fig. 8c). Comparing the ratio of Ox40 mean fluorescence intensities in cells with and without doxycycline treatment normalized to the values from cells that expressed *Ox40* constructs without 3′-UTR revealed a comparable importance of both structural elements (Fig. 6c). In fact, only deletion or point mutagenesis of the sequences encoding both structures at the same time (3′-UTR 1–80 and double mut) neutralized Roquin-dependent repression of *Ox40*. In contrast, individual mutations that left the hexaloop (3′-UTR 1–120 or CDE mut) or the CDE-like triloop intact still enabled Roquin-dependent repression, which occurred in an attenuated manner compared with the full-length 3′-UTR (Fig. 6c).

To further analyse the functional consequences of Roquin binding to the 3′-UTR, we also measured mRNA decay rates after introducing the different *Ox40* constructs into HeLa tet-off cells that allow to turn off transcription from the tetracycline-repressed vectors by addition of doxycycline (Fig. 6d). Quantitative reverse transcriptase–PCR revealed a strong stabilization of the *Ox40* mRNA by deletion of the 3′-UTR (CDS *t*_1/2_=311 min vs full-length *t*_1/2_=96 min). A comparable stabilization was achieved by combined mutation of the CDE-like and the ADE-like SLs (ADE/CDE-like mut *t*_1/2_=255 min). Individual mutations of either the ADE-like or the CDE-like SLs showed intermediate effects (ADE-like mut *t*_1/2_=170 min, CDE-like mut *t*_1/2_=167 min), respectively. These findings underscore the importance of both structural motifs and reveal that they have an additive effect on the regulation of *Ox40* mRNA expression in cells.

## Discussion

Recent structural and functional studies have provided first insight into the RNA binding of Roquin. Structures of Roquin bound to CDE SL RNAs^11^,^12^,^13^ indicated mainly shape recognition of the SL RNA in the so-called A-site of the N-terminal region of the Roquin protein with no sequence specificity, except the requirement for a pyrimidine–purine–pyrimidine triloop. Considering that the CDE RNA recognition is mostly structure specific and not sequence dependent^11^,^12^, a wide spectrum of target mRNA might be recognized by Roquin. Some evidence for this is provided by a recent study by Landthaler and colleagues^17^.

Here we have used SELEX assays to identify a novel RNA recognition motif of Roquin-1, which is present in the *Ox40* 3′-UTR and variations of which may be found in the 3′-UTRs of many other genes^17^. Our experiments show that this SELEX-derived ADE shows functional activity comparable to the previously established CDE motif. The ADE and *Ox40* ADE-like SL RNAs adopt SL folds with a hexaloop instead of a triloop. Notably, the recognition of the respective RNA-helical stem regions by the ROQ domain is identical for the triloop and hexaloop motifs. However, the U-rich hexaloops in the ADE and ADE-like SL RNAs mediate a number of additional contacts with the helix α4 and strand β3 in the ROQ domain that are absent in the triloop CDE (Fig. 3b–f). Of particular importance for the hexaloop recognition is Tyr250, which acts as a stabilizing element for the integrity of a defined loop conformation. It stacks with nucleotides in the hexaloop but not the CDE triloop (Fig. 3b,c). The functional role of Tyr250 for ADE-mediated mRNA regulation by Roquin-1 is thus explained by our experiments (Fig. 5b,c). The preference for U-rich hexaloops depends on nucleotide-specific interactions of ROQ with U10, U11 and U13 in the *Ox40* ADE-like SL. Consistent with this, loss of ROQ binding is observed on replacement of U11 and U13 by other bases (Table 2). In spite of these differences in some aspects of the RNA recognition, overall features of Roquin targets are conserved in ADE and CDE-like RNAs, namely, a crucial role of non-sequence-specific contacts to the RNA stem and mainly shape recognition of the hexa- and triloops, respectively. A unique feature of the bound RNA structure, common to both tri- and hexaloops, is the stacking of a purine base onto the closing base pair (Fig. 3b,c). Previous structural data and the results presented here therefore suggest that Roquin may recognize additional SL RNA motifs, potentially with larger loops.

Interestingly, the SELEX-derived motif resembles the U-rich motifs that were identified recently by Murakawa *et al*.^17^. In their study, several U-rich loops of various sizes were identified by crosslinking and immunoprecipitation of Roquin-1 using PAR-CLIP and the data also included sequences comprising the U-rich hexaloop identified in our present work. Most probably, the experimental setup of Murakawa *et al*.^17^ revealed both high- and low-affinity target motifs for Roquin, whereas our structural study reports on a high-affinity binding motif. Notably, Murakawa *et al*.^17^ neither found the Roquin-regulated *Ox40* nor the *Tnf* 3′-UTRs, as both genes are not expressed in HEK 293 cells. However, their newly identified U-rich target SL within the 3′-UTR of A20 mRNA supports our conclusion that Roquin can accept alternative target motifs apart from the classical CDE triloop arrangement. It remains to be seen which exact features govern the recognition of the A20 SL by Roquin.

The regulatory *cis* RNA elements in 3′-UTRs may also be targeted by additional *trans*-acting factors. We have recently identified the endonuclease Regnase-1 as a cofactor of Roquin function that shares an overlapping set of target mRNAs^8^. In another study, the overlap in targets was confirmed, but a mutually exclusive regulation was proposed based on studies in lipopolysaccharide (LPS)-stimulated myeloid cells^18^. In these cells, Roquin induced mRNA decay only for translationally inactive mRNAs, while Regnase-1-induced mRNA decay depended on active translation of the target. In CD4^+^ T cells, Ox40 does not show derepression in individual knockouts of Roquin-1 or Roquin-2 encoding genes, but is strongly induced upon combined deficiency of both genes^7^. In addition, conditional deletion of the Regnase-1-encoding gene induced Ox40 expression in these cells^19^. Whether induced decay of *Ox40* mRNA by Roquin or Regnase proteins occurs in a mutually exclusive manner at different points during T-cell activation or shows cooperative regulation will have to await a direct comparison of T cells with single, double and triple knockouts of these genes. However, in cultures of CD4^+^ T cells, Ox40 is translated on day 4–5 and is expressed much higher in T cells with combined deficiency of Roquin-1 and Roquin-2. At this time point, the short-term inducible reconstitution with WT Roquin-1 was effective to reduced Ox40 expression, demonstrating the regulation of a translationally active mRNA by Roquin-1 in T cells (Fig. 5c).

Recombinant N-terminal protein fragments of Roquin-1 or Roquin-2 bind with comparable affinity to *Ox40* mRNA in EMSAs and the 3′-UTR of *Ox40* is similarly retained by the two recombinant proteins in filter binding assays^7^. Given the almost identical RNA contacts in both paralogues, we assume a similar recognition of ADE and CDE motifs in the *Ox40* 3′-UTR by both proteins. In contrast, structural details on how Regnase-1 can interact with these SL RNAs are currently missing. Surprisingly, transcriptome-wide mapping of Regnase-1-binding sites in crosslinking and immunoprecipitation experiments identified specific triloop structures with pyrimidine–purine–pyrimidine loops in 3- to 7-nt-long stems, as well as a novel hexaloop structure in the *Ptgs2* gene. Both were required for Regnase-1-mediated repression^18^. These findings therefore raise the possibility that Regnase-1 interacts with ADE-like hexaloop structures either in a direct or indirect manner.

Nevertheless, it becomes clear that composite *cis*-elements, that is, the presence of several SLs as in *Ox40* or *Icos*^11^, could attract multiple *trans*-acting factors that may potentially co-regulate or even act cooperatively to control mRNA expression through posttranscriptional pathways of gene regulation. The novel 3′-UTR loop motif that we have identified as a *bona fide* target of Roquin now expands this multilayer mode of co-regulation. We suggest that differential regulation of mRNA expression is not only achieved through multiple regulators with individual preferences for a given motif or variants thereof, but that regulators may also identify and use distinct motifs, as long as they exhibit some basic features regarding shape, size and sequence.

The presence of distinct motifs in 3′-UTRs offers a broader variability for gene regulation by RNA *cis* elements. Their accessibility can be modulated by *trans*-acting factors that may bind regulatory motifs, unfold higher-order structures in the RNA^20^ or maintain a preference for duplex structures as was shown recently for mRNAs that are recognized by Staufen-1 (ref. 21). In the 3′-UTR of the *Ox40* mRNA, we find one ADE-like and one CDE-like SL, with similar binding to the ROQ domain. The exact stoichiometry of Roquin bound to the *Ox40* 3′-UTR is unknown. The recently identified secondary binding site for dsRNA in Roquin (B-site^12^) could potentially allow for simultaneous binding of dsRNA and thereby promote engagement of Roquin and target RNAs before recognition of high-affinity SLs. In this respect, it is interesting to note that symmetry-related RNA molecules of both *Tnf* CDE and ADE SL RNAs are found in the respective crystal lattice in a position that corresponds to the recognition of dsRNA in the B site^11^,^12^,^22^. This opens the possibility that one Roquin molecule may cluster two motifs in a given 3′-UTR and/or cluster motifs from distinct 3′-UTRs to enhance downstream processing. Interestingly, two SL RNA elements that resemble *bona fide* ligands of Roquin have also been identified in the 3′-UTR of the *Nfkbid* mRNA^9^. We therefore hypothesize that the combination of multiple binding sites may be more commonly used to enhance the functional activity of Roquin. At the same time, the combination of *cis* elements may be important for differential gene regulation, as composite *cis* elements with lower affinity may be less sensitive to Roquin. This will lead to less effective repression in T cells when antigen recognition is of moderate signal strength and only incomplete cleavage of Roquin by MALT1 occurs^8^. For understanding the intricate complexity of 3′-UTR regulation, future work will be necessary by combining large-scale approaches, such as cross-linking and immunoprecipitation experiments to identify RNA-binding sites, and structural biology to dissect the underlying molecular mechanisms^23^.

## Methods

### SELEX experiments

Selection of Roquin-1-bound RNAs from a random RNA library was performed in three rounds of selection with increased stringency of washing (3 × 100 μl, 4 × 100 μl and 5 × 100 μl washing steps) and with decreased protein concentrations (250, 150 and 50 nM). Before selection, 100 μg recombinant Roquin-1 and Roquin-1 M199R N-terminal protein (residues 2–440) were biotinylated: proteins were incubated for 30 min on ice with 10 × molar excess of EZ-link PEG4-NHS-Biotin (Pierce) in PBS (0.1 mg ml^−1^). Subsequently, the biotinylated protein was purified via gel filtration (MicroSpin column P6, BioRad) and the loss of protein during the biotinylation procedure was estimated by SDS–PAGE and Coomassie staining. The efficiency of the biotinylation reaction was evaluated after spotting of unlabelled and labelled proteins onto a nitrocellulose membrane. After blocking the membrane with 1% BSA in PBS, it was incubated in streptavidin–PE (R-Phycoerythrin) diluted 1:1,000 in PBS for 30 min at room temperature (RT). Subsequently, the membrane was washed three times with PBS and fluorescence intensity of PE bound to biotinylated protein was determined by fluoroimaging (Raytest, FLA5000, 473 nm, Y510 filter).

The RNA startpool containing the 47-nt random sequence as well as the RNA pools for the second and third selection rounds were transcribed *in vitro* from double-stranded PCR DNA, and protein-bound RNA was isolated and reverse transcribed before PCR amplification, as previously described^24^. Following transcription, the samples were separated on an 8% PAGE, the bands excised and RNA purified. Every round of selection started by combining the RNA pool (400 pmol) with biotinylated protein and incubating the mix for 30 min at 37 °C. Subsequently, binding buffer-equilibrated streptavidin-magnetic beads were added and incubated (10 min, 37 °C) to bind the protein–RNA complexes, followed by washes. By boiling the beads in 0.2 mM EDTA in water for 3 min, protein and RNA molecules were released. After removal of beads, the solution served as template for reverse transcription (One-Step RT-PCR Kit, Qiagen) and from the obtained complementary DNA the RNA pool of the next round of selection was transcribed. The cDNAs from every selection round (startpool, round 1, round 2 and round 3) were used for Index-PCRs to analyse the pool composition at every stage during selection. Comparable amounts of the PCR products were combined to one cDNA library and analysed by Solexa Illumina sequencing.

### Sequence motif and structural analysis

To identify sequence motifs to which Roquin specifically binds, we counted the number of occurrences of each hexamer (4^6^=4,096 motifs) in the sequences obtained by SELEX. We then generated a data set of randomized sequences of the same nucleotide composition as the SELEX-derived sequences, by permuting the SELEX-derived sequences with a custom script. Finally, we counted the number of occurrences of each hexamer in the set of randomized sequences and computed the log_2_ ratio of the number of occurrences of each motif in the real and randomized sequence sets. To identify a shared sequence motif in the SELEX patterns that showed the strongest enrichment in our selection experiments, the top 100 patterns were analysed with the Motif-based sequence analysis tool MEME 25 (http://meme-suite.org) using the default settings. This analysis revealed three sequence motifs of which the first is shown in Fig. 1b. For the construction of sequence logos, we screened the obtained nucleotide sequences from SELEX replicate 1 and extracted the nucleotide sequences including the 7-nt flanking regions. Sequence logos were constructed with WebLogo 2.8.2 (http://weblogo.berkeley.edu/).

For the *Ox40* 3′-UTR sequence alignment, we extracted Multiz alignments^26^ of 60 Vertebrates from the UCSC mouse GRCm38/mm10 assembly for the genomic region chr4:156,016,498–156,016,520. For each species contained in the alignment, we extracted genomic coordinates of the aligned sequence, extended the coordinates by 10 nt upstream and downstream, and retrieved the extended sequences from the corresponding genome assemblies. Finally, sequences were aligned with ClustalW 2.1 with standard settings and the alignment was visualized using Jalview.

To evaluate the structural context the inferred motif is located in, we first appended to the nucleotide sequences obtained from the SELEX experiment the SELEX primers 5′-GGAGAGATGTGAACTT-3′ and 5′-AGTTTCGTGGATGCCAC-3′ to the 5′- and 3′-end, respectively. Next, we screened for sequences that contained the inferred motif and performed secondary structure prediction on those sequences with RNAfold from the ViennaRNA package version 1.8 with parameters '-p -d2'. Next, we used a custom Perl script to parse the base-pairing probability file generated by RNAfold and to calculate an average base-pair probability over all sequences that contained the inferred motif.

### Production of proteins

Cloning of expression vectors for Roquin-1 ROQ (residues 147–326), ROQ (residues 171–326) and Roquin-1 N-term (residues 2–440) was carried out by standard procedures as described previously^11^. Briefly, PCR-amplified fragments were put into pETM11 and pETTrx1a vectors based on pET24d as provided by the Protein Expression and Purification Facility at Helmholtz Zentrum München. All vectors contained tobacco etch virus (TEV) protease recognition sites for subsequent proteolytic removal of the tags. All length-variable Roquin-1 expression constructs were designed and cloned via restriction sites NcoI (5′) and XhoI (3′). ROQ domain RNA-binding mutants were cloned by Quick change PCR with high-fidelity Phusion DNA polymerase and subsequent treatment with DnpI. Alternatively, we used conventional cloning with a two-step PCR protocol and enzymatic restriction.

The Roquin-1 fragments (147–326) and (171–326) were expressed as N-terminal His_6_-thioredoxin fusion proteins as recently described^11^. Isotope-labelled protein for NMR studies was expressed in M9 minimal medium supplemented with 0.5 g l^−1 15^N ammonium chloride and 2 g l^−1^unlabelled or [U-^13^C] glucose. For the preparation of deuterated proteins, cells were adapted and grown as described previously^27^. Briefly, we used a protocol with stepwise adaptation of cells to deuterium changing buffer from no D_2_O, low glucose to 50% D_2_O, low glucose and finally 99.5% D_2_O with deuterated glucose. The Roquin-1 N-terminal domain (residues 2–440) was expressed and purified essentially as described above for the ROQ domain, but no thioredoxin tag was used. For Roquin-1 N-terminal domain, all expression media and the final buffer contained 100 or 25 μM of zinc chloride, respectively.

### RNA preparation

RNAs were synthesized and purchased from IBA GmbH (Göttingen, Germany), purified via PAGE followed by two steps of desalting. No major impurities were seen in NMR spectra. Complex formation for crystallography and NMR experiments was achieved by dissolving the lyophilized RNA in water or NMR buffer. This stock solution was snap-cooled by boiling at 95 °C for 5 min and transferred to an ice-cold bath for 10 min before aliquoting. All RNAs were stored at −80 °C, to avoid degradation and thermodynamically favoured duplex formation.

Full length and fragments of *Ox40* 3′-UTR mRNA were produced by *in vitro* transcription (IVT) from DNA templates harbouring a T7 promoter site either with direct incorporation of α-^32^P-labelled UTP or subsequent 3′-labelling of purified RNA with γ-^32^P-labelled ATP. DNA templates were cloned by primer extension PCR. For IVT, 50–150 nM of DNA were incubated with 11 mM magnesium chloride, 8% (w/v) PEG8000, 1.25 mM of each NTP and 0.05 mg ml^−1^ of T7 polymerase in 1 × standard reaction buffer for 3–5 h at 37 °C. Labelled RNAs were produced in 50 μl reactions and purified via spin columns and directly subjected to EMSA assays. Unlabelled RNAs were produced in reactions of 500–5,000 μl. After IVT, the reactions were separated on 8% denaturing SDS–PAGEs, RNA of interest excised and eluted from the gel using the Elutrap kit (GE Healthcare). After elution, RNAs were dialysed against water and lyophilized. Subsequently, RNAs were dissolved in water and stocks generated by boiling them at 95 °C for 5 min and transferred to an ice-cold bath for 10 min before aliquoting. Labelling for EMSA assays was carried out as for short motifs and described recently^11^. As a modification, dephosphorylation was performed for 30 min and 3′-phosphorylation with γ-^32^P-labelled ATP and T4 polynucleotide kinase for 90 min for higher efficiency, respectively.

### NMR spectroscopy

NMR measurements of Roquin-1 ROQ (147–326) and ROQ (171–326) were performed in buffers as described, mixed with 10% D_2_O. Backbone chemical shift assignments of ROQ (171–326) with 1.1- to 1.2-fold excess of the *Ox40 ADE-like* SL motif or consensus ADE SL RNAs were recorded with protein concentrations of 350–400 μM. HNCA, HNCACB, HNCO, HNcaCO and 3D ^15^N-edited NOESY spectra^28^ were acquired at 298K on Bruker Avance III spectrometers at field strengths corresponding to 600 and 800 MHz proton Larmor frequency, equipped with TCI cryogenic probe heads. Spectra of ROQ in complex with Ox40 CDE-like SL RNA and the RNA alone have been reported before^11^. Spectra were processed with Topspin3.2 and analysed with CCPNMR Analysis^29^ and Sparky^30^. For RNA motifs, one- and two-dimensional imino NOESY spectra with water-flip-back WATERGATE were recorded at 600–900 MHz, at 278 and 298 K at 150–350 μM RNA concentrations. Sequential assignments were guided by secondary structure predictions with *mfold*^31^ and supported by ^15^N chemical shifts from natural abundance SOFAST-HMQC experiments^32^.

### Electrophoretic mobility shift assays

The EMSAs with ROQ domain and individual motifs were performed as described previously^11^. In short, for the binding reaction a mastermix containing transfer RNA, ^32^P-labelled SL RNA and reaction buffer was prepared and then mixed with dilutions of the recombinant proteins to achieve the indicated protein concentrations. The binding was performed for 10 min at RT or 20 min on ice in 20 μl reaction volume in the presence of 2.5 μg μl^−1^ tRNA from baker’s yeast (Sigma), 500 pM ^32^P-labelled RNA, 20 mM HEPES (pH 7.4), 50 mM NaCl, 1 mM MgCl_2_, 1 mM dithiothreitol and 1 μg μl^−1^ BSA. For the binding reaction of Roquin-1 N-terminal with full-length *Ox40* 3′-UTRs or fragments thereof, ∼1 pmol of RNA was incubated with protein concentrations between 0 and 1,000 μM in a volume of 20 μl. RNP complexes were resolved by PAGE (6% polyacrylamide, 5% glycerol, 0.5 × TBE) at 120 V for 40 min at RT. Gels were then fixed, dried and exposed to a phosphor imager screen.

### X-ray crystallography

The crystallization experiments for ROQ–RNA complexes were performed at the X-ray Crystallography Platform at Helmholtz Zentrum München. The crystals of both, Roquin-1 ROQ (171–326) with *Ox40* ADE-like SL motif (22mer, 5′-UCCACACCGUUCUAGGUGCUGG-3′) and with the consensus SELEX-derived ADE SL motif (20mer, 5′-UGACUGCGUUUUAGGAGUUA-3′) were obtained from the same condition: 100 mM Bis-Tris buffer pH 5.5, 200 mM sodium chloride and 25% (v/w) PEG 3350. Crystallization was performed using the sitting-drop vapour-diffusion method at 292 K in 24-well plates and a protein concentration of 12 mg ml^−1^. The crystals appeared after 1 day. For the X-ray diffraction experiments, the crystals of both co-complexes were mounted in a nylon fibre loop and flash cooled to 100 K in liquid nitrogen. The cryoprotection was performed for 2 s in reservoir solution complemented with 20% (v/v) ethylene glycol. Diffraction data for ROQ *Ox40* ADE-like motif was collected on the ID29 beamline (ESRF, Grenoble, France) using a Pilatus 6M at a wavelength of 1.25363 Å. Diffraction data for the ROQ-ADE complex were collected using Pilatus 2M detector at 1.00003 Å wavelength at PXIII beamline at SLS (Villigen, Switzerland). All data sets were indexed and integrated using *XDS*^33^ and scaled using *SCALA*^34^,^35^. Intensities were converted to structure–factor amplitudes using the programme *TRUNCATE*^36^. Table 1 summarizes data collection and processing statistics for both data sets.

### Structure determination and refinement

The structure of both ROQ-*Ox40* ADE-like SL and ROQ-ADE SL were solved by molecular replacement using the native Roquin-1 ROQ (147–326) structure as a search model (PDB: 4QI0 (ref. 11)). Model building was performed in *COOT*^37^. RNA molecules were modelled manually. The refinement of both structures was done in *REFMAC5* (ref. 38) using the maximum-likelihood target function including translation, libration and screw-rotation displacements of a pseudo-rigid body (TLS)^39^. For the ROQ-ADE SL structure, non-crystallographic symmetry (NCS) averaging was implemented. The final models are characterized by *R* and *R*_free_ factors of 21.8 and 25.7% for ROQ-*Ox40* ADE-like SL, and 18.6 and 23.4% for ROQ-ADE SL (Table 1), respecively. The stereochemical analysis of both final models was done in *PROCHECK*^40^ and *MolProbity*^41^. It indicates that there are no residues with generously allowed or unfavourable backbone dihedral angles, and that 99.4% (for ROQ-*Ox40* ADE-like SL structure) and 92.3% (for ROQ-ADE SL structure) of all residues are in the core region of the Ramachandran plot.

### Functional assays

Functional assays determining the Roquin-mediated regulation of *Ox40* with different 3′-UTR variants were performed as described previously^8^. In brief, *Rc3h1/2*^*−/−*^ mouse embryonic fibroblast (MEF) cells, stably transduced with a doxycycline-inducible Roquin-1-p2A-mCherry construct, were retrovirally infected with *Ox40* constructs of different 3′-UTR length or mutation, which led to the expression of Ox40 on the cell surface (CDE-like mutation changing nt 14–16 GCA to CGT, ADE-like mutation changing nt 15–17 from GGT to CCA). Forty-eight hours after infection, the cells were split and one half of the cells was treated with doxycycline (1 μg ml^−1^), to induce expression of Roquin-1 and mCherry, connected via the self-cleaving peptide p2A. Thus, Roquin-expressing cells were marked by mCherry expression. Sixteen to 20 h after induction, the cells were harvested, stained with allophycocyanin (APC)-conjugated anti-Ox40 and analysed by flow cytometry. To compare the Ox40 expression levels achieved by different constructs, the relative Ox40 mean fluorescence intensity (MFI) was determined by dividing the MFI of treated (mCherry^+^) cells by the MFI of untreated (mCherry^−^) cells.

### Mouse experiments

Compound mutant mice with the *Rc3h1*^fl/fl^ (ref. 4) and *Rc3h2*^fl/fl^ (ref. 7) (denoted *Rc3h1/2*^fl/fl^), as well as *Cd4*-Cre-ERT2 (ref. 42) and *Gt(ROSA)26Sor*^tm1(rtTA*M2)Jae^ alleles were maintained on a C57BL/6 genetic background. All animals were housed in a pathogen-free barrier facility in accordance with the Ludwig-Maximilians-University München institutional, state and federal guidelines.

### Generation of overexpression vectors

Expression constructs of Roquin-1 and Ox40 were cloned into a modified pRetroX-Tight vector (Clontech). The puromycine-resistance cassette was removed and a cassette containing attR1-ccdB-attR2 was inserted, to generate a Gateway destination vector. Roquin-1 and Ox40 constructs were inserted by LR reaction (Invitrogen). Any mutants thereof were generated by site-directed mutagenesis.

### Virus production

Replication-deficient retrovirus production and T-cell transduction was performed as previously described^7^,^43^. Briefly, retroviral and packaging plasmids were introduced into HEK293T cells by calcium-phosphate transfection. Forty-eight hours after transfection, cell culture supernatants containing the retrovirus particles were harvested, passed through 0.45-μm filters and stored at **−**80 °C.

### Cell isolation and culture

Splenocytes were isolated from *Rc3h1/2*^fl/fl^; *Cd4*-Cre-ERT2; rtTA mice. CD4^+^ T cells were isolated by negative selection with magnetic beads according to the manufacturer’s instructions (Stem Cell Technologies). CD4^+^ T cells were cultured in DMEM medium supplemented with 10% (vol/vol) fetal bovine serum, 1 × nonessential amino acids (Lonza), 10 mM HEPES pH 7.4 (Invitrogen), 50 μM β-mercaptoethanol (Invitrogen) and 100 U ml^−1^ penicillin–streptomycin (Invitrogen). *Rc3h1/2*^fl/fl^ deletion was induced by addition of 4′OH-Tamoxifen (0.3 μM) for 24 h. For T_H_1 differentiation, CD4^+^ T cells were cultured in six-well plates pre-coated with goat anti-hamster IgG (MP Biochemicals) and DMEM medium further supplemented with anti-CD3 (0,25 μg ml^−1^), anti-CD28 (2,5 μg ml^−1^), IL-12 (10 ng ml^−1^) and anti-IL-4 (10 μg ml^−1^) for 40 h. Cells were then infected with retroviral constructs, allowing reconstitution with either Roquin-1, Roquin-1 Y250A or Roquin-1 K220A, K239A and R260A, and cultured in IL-2 containing media (20 U ml^−1^). Forty-eight hours after transduction, the cells were split and one half of cells was treated with doxycycline (1 μg ml^−1^), to induce expression of Roquin-1 WT and Roquin-1 mutants. Twenty-four hours after induction, the cells were harvested for analysis by immunoblot and flow cytometry with the indicated antibodies (1:200 anti-mouse Icos-biotin clone 7E–17G9 (eBioscience); 1:200 Streptavidin-PerCP (Becton Dickinson); 1:200 anti-mouse Ox40-PE clone OX-86 (eBioscience)).

### Immunoblot analysis

CD4^+^ T cells were incubated for 15 min on ice with lysis buffer (20 mM Tris-HCl pH 7.5, 150 mM NaCl, 0.25% (vol/vol) Nonidet-P40, 1.5 mM MgCl_2_ and protease inhibitor mix without EDTA (Roche) and 1 mM dithiothreitol). Lysate was cleared by centrifugation (10 min, 10 000 *g*, 4 °C). Immunoblotting was performed by standard protocols with hybridoma supernatants containing monoclonal antibody recognizing Roquin-1 and Roquin-2 (anti-roquin, clone 3F12)^7^.

### mRNA decay experiments

Hela Tet-Off Advanced Cells (Clontech 631156) were stably transduced with retroviruses expressing different Ox40 constructs. FACS analysis 41 h post transduction revealed similar Ox40 surface expression levels on all five cell samples. After transduction, the cell lines were initially cultured for at least 48 h without doxycycline, to ensure high Ox40-expression levels. For each time point, 400 000 cells were spread on one well in a six-well plate. To switch off Ox40-transcription, doxycycline was supplied with the medium at time point 0. After one washing step with PBS, cells were directly harvested from each well with Trizol before Dox application (0 h), as well as 2, 3 and 4 h after Dox application. RNA was isolated using standard Trizol protocols. Reverse transcription was performed with the Qiagen Quantitect Reverse Transcription Kit following the manufacturer’s protocols. Quantitative PCR was carried out on a Roche Light Cycler 480 using the Light Cycler 480 Probes Master Mix and primer-/probe-combinations from Roches Universal Probe Library. Relative mRNA expression levels were calculated by normalization to the housekeeper gene *ywhaz*.

### Surface plasmon resonance

ROQ–RNA binding experiments were performed on a BIACORE 3000 instrument (Biacore Inc.). ROQ domain was diluted to a final concentration of 35 μg ml^−1^ in 10 mM HEPES pH 7.0 and chemically immobilized (amine coupling) onto CM5 sensor chips (Biacore Inc.). The RNA samples were diluted in the running buffer (10 mM HEPES pH 7.4, 150 mM NaCl, 2 mM MgCl_2_ and 0.005% Tween 20) to the final concentration of 31.25, 62.5, 125, 250 and 500 nM, and 1 and 2 μM, and injected over the sensor chip surface at 30 μl min^−1^ at 10 °C. The RNA samples were injected onto the sensor chip from the lowest to the highest concentration. Each RNA-type sample was tested three times with the exception of Mut1–3 two times. Injection of 250 nM RNA was always performed in duplicate within each experiment. To subtract any background noise from each data set, all samples were also run over an unmodified sensor chip surface. Data were analysed using BIAevaluation programme (Biacore Inc.) (Supplementary Fig. 7). For each measurement, the equilibrium dissociation constant was calculated (*K*_D_) from steady state binding. The *K*_D_ from three independent experiments were used to calculate the mean values of these variables and the s.e.m. The results for all tested RNA samples are compared in Table 2.

## Additional information

**Accession codes:** Atomic coordinates and structure factors have been deposited in the Protein Data Bank under accession codes 5F5H and 5F5F for the ROQ-*Ox40* ADE-like SL and ROQ-ADE SL, respectively. Chemical shifts of the ROQ-*Ox40* ADE-like SL and ROQ-ADE SL have been deposited in the Biological Magnetic Resonance Data Bank under accession codes 26587 and 26588, respectively.

**How to cite this article:** Janowski, R. *et al*. Roquin recognizes a non-canonical hexaloop structure in the 3′-UTR of *Ox40*. *Nat. Commun.* 7:11032 doi: 10.1038/ncomms11032 (2016).

## Supplementary Material

Supplementary Information Supplementary Figures 1-8, Supplementary Notes and Supplementary Reference.

## Figures and Tables

**Table 1 t1:** Data collection and refinement statistics.

	**ROQ-*****Ox40*** **ADE-like SL**	**ROQ-ADE SL**
*Data collection*		
space group	*P*2_1_2_1_2	*P*2_1_2_1_2_1_
		
Cell dimensions		
*a*, *b*, *c* (Å)	89.66, 115.79, 42.61	72.90, 89.30, 144.70
*α, β, γ* (°)	90, 90, 90	90, 90, 90
Resolution (Å)	50–2.23 (2.29–2.23)	50–3.0 (3.08–3.00)
*R*_merge_	5.9 (68.3)	14.8 (93.8)
*I*/σ*I*	14.9 (2.1)	16.7 (3.1)
Completeness (%)	98.7 (97.7)	99.9 (99.9)
Redundancy	3.9 (3.7)	13.2 (12.7)
		
*Refinement*		
Resolution (Å)	2.23	3.00
No. reflections	21,018	18,598
*R*_work_/*R*_free_	21.8/25.7	18.6/23.4
		
No. atoms		
Protein	2,404	4,820
Ligand/ion	894	1,708
Water	99	49
* B*-factor overall	47.2	60.4
		
*Root mean squared deviations*		
Bond lengths (Å)	0.006	0.014
Bond angles (°)	1.07	1.77
		
Ramachandran plot		
Most favoured (%)	98.6	99.8
Additional allowed (%)	1.4	0.2

ADE, alternative decay element; CDE, constitutive decay element; SL, stem loop.

For each data set, only one crystal has been used.

*Values in parentheses are for highest-resolution shell.

**Table 2:**
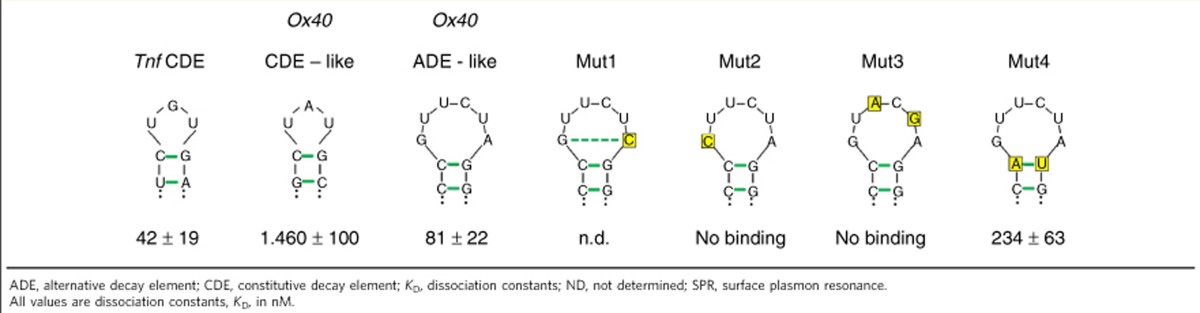
*K*_D_ for selected RNAs obtained from SPR measurements with immobilized ROQ domain of Roquin-1.

## References

[b1] Turner M. & Hodson D. Regulation of lymphocyte development and function by RNA-binding proteins. Curr. Opin. Immunol. 24, 160–165 (2012). 22326859 10.1016/j.coi.2012.01.011

[b2] Kafasla P., Skliris A. & Kontoyiannis D. L. Post-transcriptional coordination of immunological responses by RNA-binding proteins. Nat. Immunol. 15, 492–502 (2014). 24840980 10.1038/ni.2884

[b3] Vinuesa C. G. . A RING-type ubiquitin ligase family member required to repress follicular helper T cells and autoimmunity. Nature 435, 452–458 (2005). 15917799 10.1038/nature03555

[b4] Bertossi A. . Loss of Roquin induces early death and immune deregulation but not autoimmunity. J. Exp. Med. 208, 1749–1756 (2011). 21844204 10.1084/jem.20110578 PMC3171092

[b5] Heissmeyer V. & Vogel K. U. Molecular control of Tfh-cell differentiation by Roquin family proteins. Immunol. Rev. 253, 273–289 (2013). 23550652 10.1111/imr.12056

[b6] Pratama A. . Roquin-2 shares functions with its paralog Roquin-1 in the repression of mRNAs controlling T follicular helper cells and systemic inflammation. Immunity 38, 669–680 (2013). 23583642 10.1016/j.immuni.2013.01.011

[b7] Vogel K. U. . Roquin paralogs 1 and 2 redundantly repress the Icos and Ox40 costimulator mRNAs and control follicular helper T cell differentiation. Immunity 38, 655–668 (2013). 23583643 10.1016/j.immuni.2012.12.004

[b8] Jeltsch K. M. . Cleavage of roquin and regnase-1 by the paracaspase MALT1 releases their cooperatively repressed targets to promote T(H)17 differentiation. Nat. Immunol. 15, 1079–1089 (2014). 25282160 10.1038/ni.3008

[b9] Leppek K. . Roquin promotes constitutive mRNA decay via a conserved class of stem-loop recognition motifs. Cell 153, 869–881 (2013). 23663784 10.1016/j.cell.2013.04.016

[b10] Yu D. . Roquin represses autoimmunity by limiting inducible T-cell co-stimulator messenger RNA. Nature 450, 299–303 (2007). 18172933 10.1038/nature06253

[b11] Schlundt A. . Structural basis for RNA recognition in roquin-mediated post-transcriptional gene regulation. Nat. Struct. Mol. Biol. 21, 671–678 (2014). 25026077 10.1038/nsmb.2855

[b12] Tan D., Zhou M., Kiledjian M. & Tong L. The ROQ domain of Roquin recognizes mRNA constitutive-decay element and double-stranded RNA. Nat. Struct. Mol. Biol. 21, 679–685 (2014). 25026078 10.1038/nsmb.2857 PMC4125485

[b13] Sakurai S., Ohto U. & Shimizu T. Structure of human Roquin-2 and its complex with constitutive-decay element RNA. Acta Crystallogr. Sect. F Struct. Biol. Commun. 71, (Pt 8): 1048–1054 (2015). 26249698 10.1107/S2053230X15011887 PMC4528940

[b14] Gewies A. . Uncoupling Malt1 threshold function from paracaspase activity results in destructive autoimmune inflammation. Cell Rep. 9, 1292–1305 (2014). 25456129 10.1016/j.celrep.2014.10.044

[b15] Athanasopoulos V. . The ROQUIN family of proteins localizes to stress granules via the ROQ domain and binds target mRNAs. FEBS J. 277, 2109–2127 (2010). 20412057 10.1111/j.1742-4658.2010.07628.x

[b16] Schlundt A. . A xenon-129 biosensor for monitoring MHC-peptide interactions. Angew. Chem. Int. Ed. Engl. 48, 4142–4145 (2009). 19408266 10.1002/anie.200806149

[b17] Murakawa Y. . RC3H1 post-transcriptionally regulates A20 mRNA and modulates the activity of the IKK/NF-kappaB pathway. Nat. Commun. 6, 7367 (2015). 26170170 10.1038/ncomms8367 PMC4510711

[b18] Mino T. . Regnase-1 and Roquin regulate a common element in inflammatory mRNAs by spatiotemporally distinct mechanisms. Cell 161, 1058–1073 (2015). 26000482 10.1016/j.cell.2015.04.029

[b19] Uehata T. . Malt1-induced cleavage of regnase-1 in CD4(+) helper T cells regulates immune activation. Cell 153, 1036–1049 (2013). 23706741 10.1016/j.cell.2013.04.034

[b20] Rouskin S., Zubradt M., Washietl S., Kellis M. & Weissman J. S. Genome-wide probing of RNA structure reveals active unfolding of mRNA structures in vivo. Nature 505, 701–705 (2014). 24336214 10.1038/nature12894 PMC3966492

[b21] Sugimoto Y. . hiCLIP reveals the in vivo atlas of mRNA secondary structures recognized by Staufen 1. Nature 519, 491–494 (2015). 25799984 10.1038/nature14280 PMC4376666

[b22] Schlundt A., Niessing D., Heissmeyer V. & Sattler M. RNA recognition by Roquin in post-transcriptional gene regulation. Wiley Interdiscip Rev RNA in press doi:; DOI: 10.1002/wrna.1333 (2015). 26844532

[b23] Hennig J. & Sattler M. Deciphering the protein-RNA recognition code: combining large-scale quantitative methods with structural biology. BioEssays 37, 899–908 (2015). 26059946 10.1002/bies.201500033

[b24] Chang T. W. . *In vitro* selection of RNA aptamers that inhibit the activity of type A botulinum neurotoxin. Biochem. Biophys. Res. Commun. 396, 854–860 (2010). 20452328 10.1016/j.bbrc.2010.05.006 PMC2891020

[b25] Bailey T. L. & Elkan C. Fitting a mixture model by expectation maximization to discover motifs in biopolymers. Proc. Int. Conf. Intell. Syst. Mol. Biol. 2, 28–36 (1994). 7584402

[b26] Blanchette M. . Aligning multiple genomic sequences with the threaded blockset aligner. Genome Res. 14, 708–715 (2004). 15060014 10.1101/gr.1933104 PMC383317

[b27] Günther S. . Bidirectional binding of invariant chain peptides to an MHC class II molecule. Proc. Natl Acad. Sci. SA 107, 22219–22224 (2010). 10.1073/pnas.1014708107 PMC3009805 21115828

[b28] Sattler M., Schleucher J. & Griesinger C. Heteronuclear multidimensional NMR experiments for the structure determination of proteins in solution employing pulsed field gradients. Prog. NMR Spectrosc. 34, 93–158 (1999).

[b29] Vranken W. F. . The CCPN data model for NMR spectroscopy: development of a software pipeline. Proteins 59, 687–696 (2005). 15815974 10.1002/prot.20449

[b30] Goddard T. D. & Kneller D. G. SPARKY 3 Univ. California, San Francisco.

[b31] Zuker M. Mfold web server for nucleic acid folding and hybridization prediction. Nucleic Acids Res. 31, 3406–3415 (2003). 12824337 10.1093/nar/gkg595 PMC169194

[b32] Schanda P. & Brutscher B. Very fast two-dimensional NMR spectroscopy for real-time investigation of dynamic events in proteins on the time scale of seconds. J. Am. Chem. Soc. 127, 8014–8015 (2005). 15926816 10.1021/ja051306e

[b33] Kabsch W. Xds. Acta Crystallogr. D Biol. Crystallogr. 66, (Pt 2): 125–132 (2010). 20124692 10.1107/S0907444909047337 PMC2815665

[b34] Evans P. Scaling and assessment of data quality. Acta Crystallogr. D Biol. Crystallogr. 62, (Pt 1): 72–82 (2006). 16369096 10.1107/S0907444905036693

[b35] Winn M. D. . Overview of the CCP4 suite and current developments. Acta Crystallogr. D Biol. Crystallogr. 67, (Pt 4): 235–242 (2011). 21460441 10.1107/S0907444910045749 PMC3069738

[b36] French S. & Wilson K. On the treatment of negative intensity observations. Acta Crystallogr. A 34, 517–525 (1978).

[b37] Emsley P., Lohkamp B., Scott W. G. & Cowtan K. Features and development of Coot. Acta Crystallogr. D Biol. Crystallogr. 66, (Pt 4): 486–501 (2010). 20383002 10.1107/S0907444910007493 PMC2852313

[b38] Murshudov G. N., Vagin A. A. & Dodson E. J. Refinement of macromolecular structures by the maximum-likelihood method. Acta Crystallogr. D Biol. Crystallogr. 53, (Pt 3): 240–255 (1997). 15299926 10.1107/S0907444996012255

[b39] Winn M., Isupov M. & Murshudov G. N. Use of TLS parameters to model anisotropic displacements in macromolecular refinement. Acta Crystallogr. D Biol. Crystallogr. 57, 122–133 (2000). 10.1107/s0907444900014736 11134934

[b40] Laskowski R., MacArthur M. W., Moss D. S. & Thornton J. M. PROCHECK: a program to check the stereochemical quality of protein structures. J. Appl. Cryst. 26, 283–291 (1993).

[b41] Chen V. B. . MolProbity: all-atom structure validation for macromolecular crystallography. Acta Crystallogr. D Biol. Crystallogr. 66, (Pt 1): 12–21 (2010). 20057044 10.1107/S0907444909042073 PMC2803126

[b42] Sledzinska A. . TGF-beta signalling is required for CD4(+) T cell homeostasis but dispensable for regulatory T cell function. PLoS Biol. 11, e1001674 (2013). 24115907 10.1371/journal.pbio.1001674 PMC3792861

[b43] Glasmacher E. . Roquin binds inducible costimulator mRNA and effectors of mRNA decay to induce microRNA-independent post-transcriptional repression. Nat. Immunol. 11, 725–733 (2010). 20639877 10.1038/ni.1902

