# Supplementary material for: Roquin recognizes a non-canonical hexaloop structure in the 3′-UTR of Ox40
Source: Nat Commun. 2016 Mar 24;7:11032. doi: 10.1038/ncomms11032 (PMC5603727; doi:10.1038/ncomms11032)
Supplement: Supplementary Information — Supplementary Figures 1-8, Supplementary Notes and Supplementary Reference. [file ncomms11032-s1.pdf]

Supplementary Fig. 1

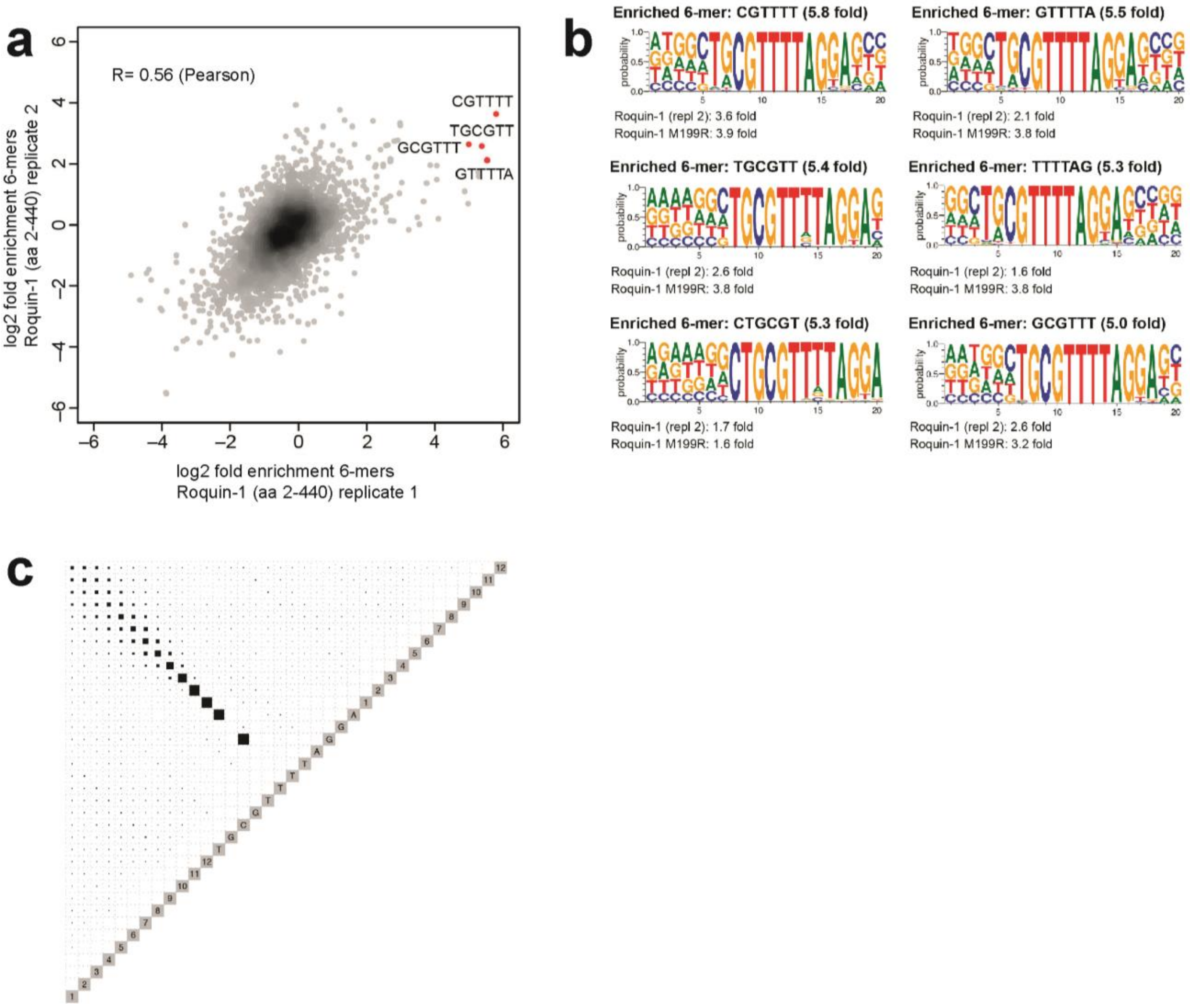

SELEX identifies a Roquin-1-recognized hexa-loop structure. (**a**) Correlation analysis of hexamers enriched by Roquin-1 (residues 2-440) in two independent SELEX experiments. The most frequent hexamers in both samples are shown in red. (**b**) The enriched hexamers that were found by Roquin-1 N-terminus (residues 2-440) or Roquin-1 M199R N-terminus (residues 2-440) are embedded in very similar sequences. (**c**) Analysis of the structural context of the SELEX-derived motif. The area of the black boxes is proportional to the probability that nucleotides at the two positions in a sequence form a base-pair. Our analysis clearly indicates that the motif GTTTTA is located in the loop of a hairpin structure.

Supplementary Fig. 2

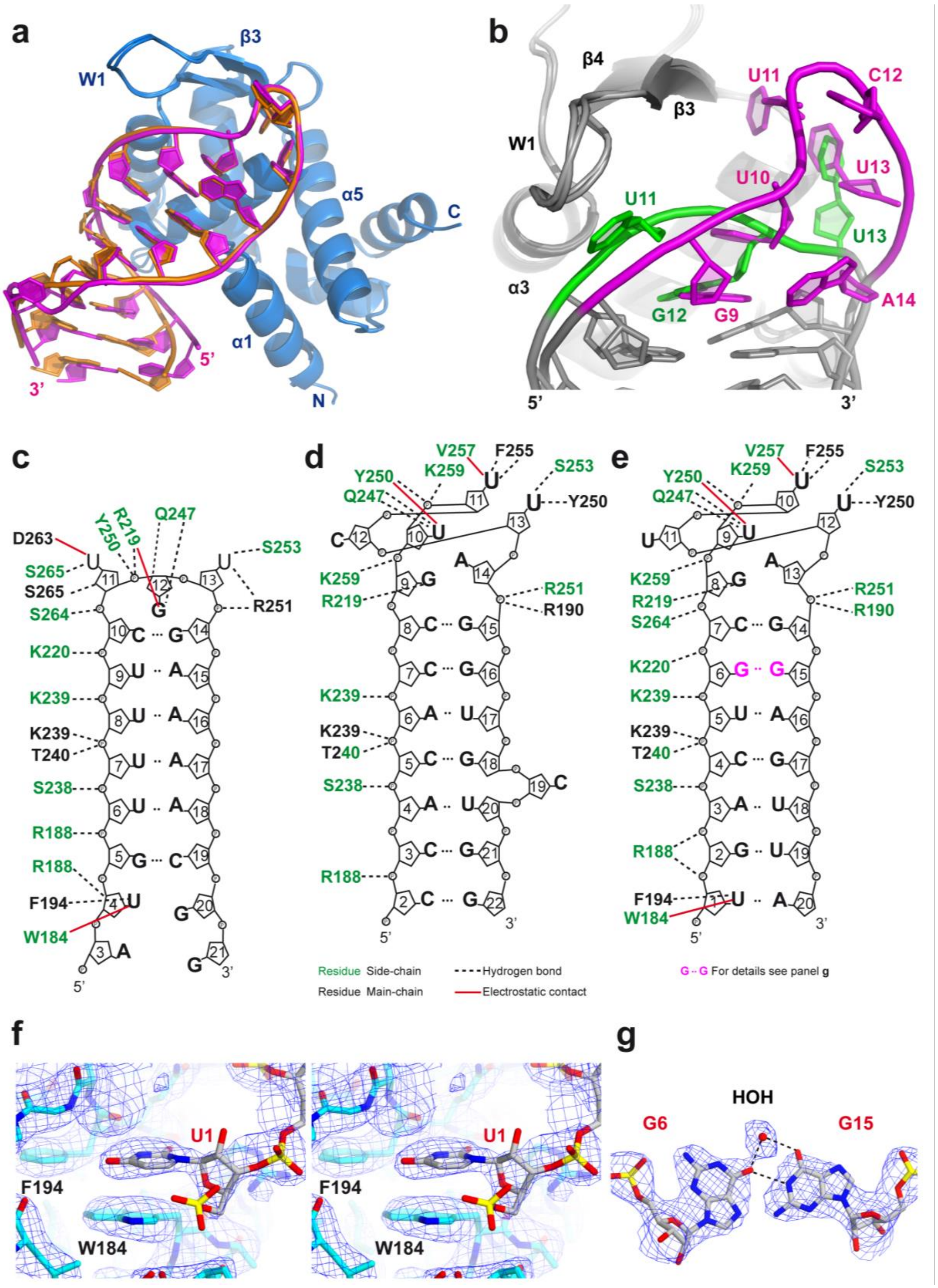

(**a**) Superposition of the Roquin-1 ROQ domain with *Ox40* ADE-like RNA (RNA shown in magenta) and ROQ-ADE (RNA shown in orange). The protein chain is shown as a blue cartoon. Selected secondary structure elements of ROQ have been labeled. The r.m.s.d. value for the superposition is 0.5 Å. (**b**) Close-up view of the superposition of the hexa-loop from *Ox40* ADE-like RNA (RNA shown in magenta) and *Tnf* CDE SL RNA (in green) bound to theRoquin-1 ROQ domain. Selected nucleotides and protein secondary structure elements are labeled. (**c-e**) Schematic drawings of the protein-RNA interactions and RNA-RNA base-pairing observed in the ROQ–*Tnf* CDE SL, ROQ-*Ox40* ADE-like SL and ROQ-ADE SL complex structures, respectively. (**f**) Stereo view of the 2Fo-Fc electron density map (1σ contour) showing the interactions of U1 with W184 and F194 in the ROQ-ADE SL structure. (**g**) The 2Fo-Fc electron density map (1σ contour) for the G6-G15 pairing in the ROQ-ADE SL complex structures.

Supplementary Fig. 3

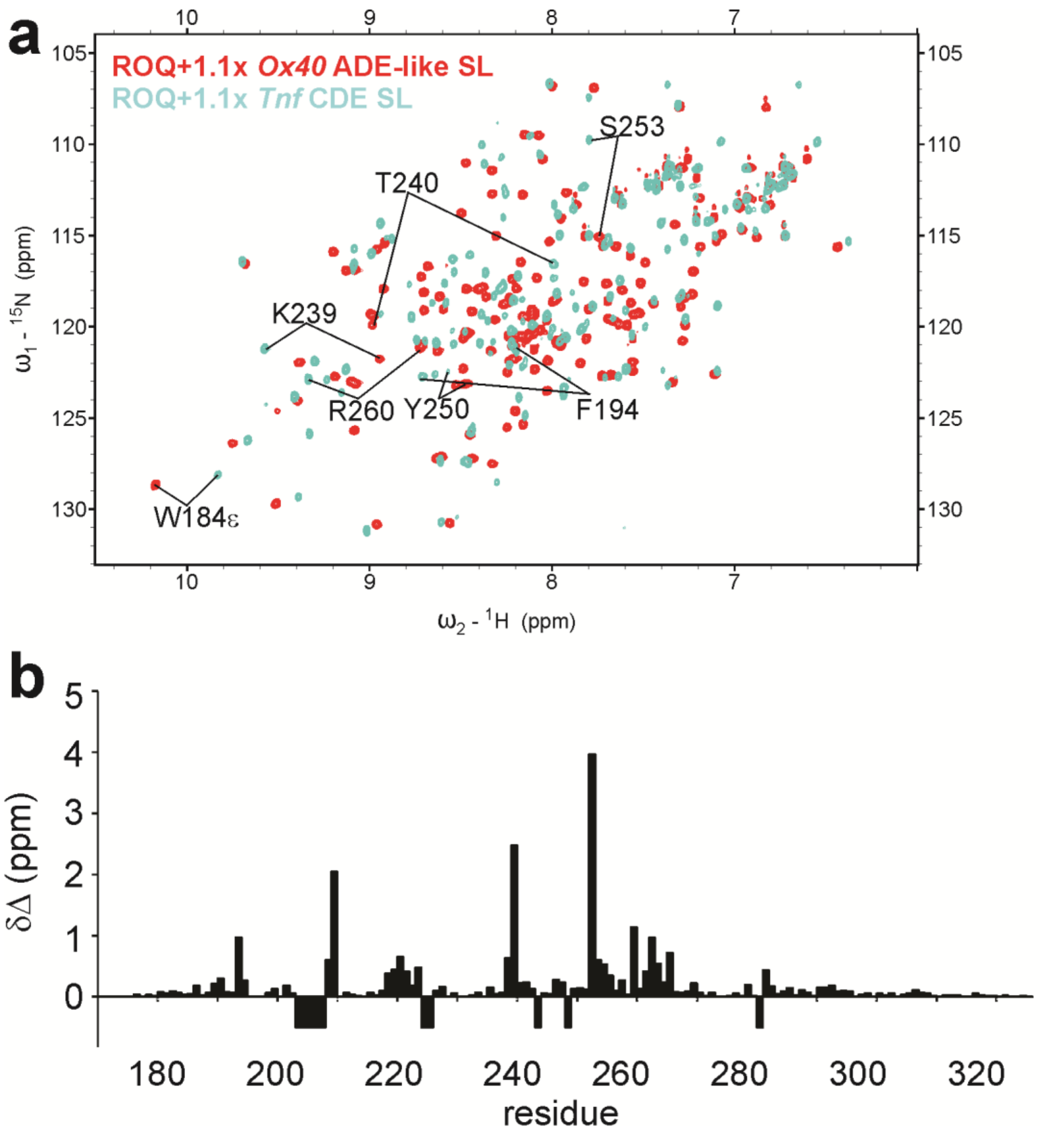

(**a**) Overlay of ^{1}H-^{15}N-HSQC spectra of either the ROQ domain in complex with the *Ox40* ADE-like SL (red) or the *Tnf* CDE SL (cyan). (**b**) Plot of CSPs comparing the Roquin-1 ROQ (171-326) domain in complex with the mRNA motifs as shown by HSQCs in (a). Negative bars in indicate missing assignments in one of the complexes and gaps derive from prolines. Selected assignments are shown.

Supplementary Fig. 4

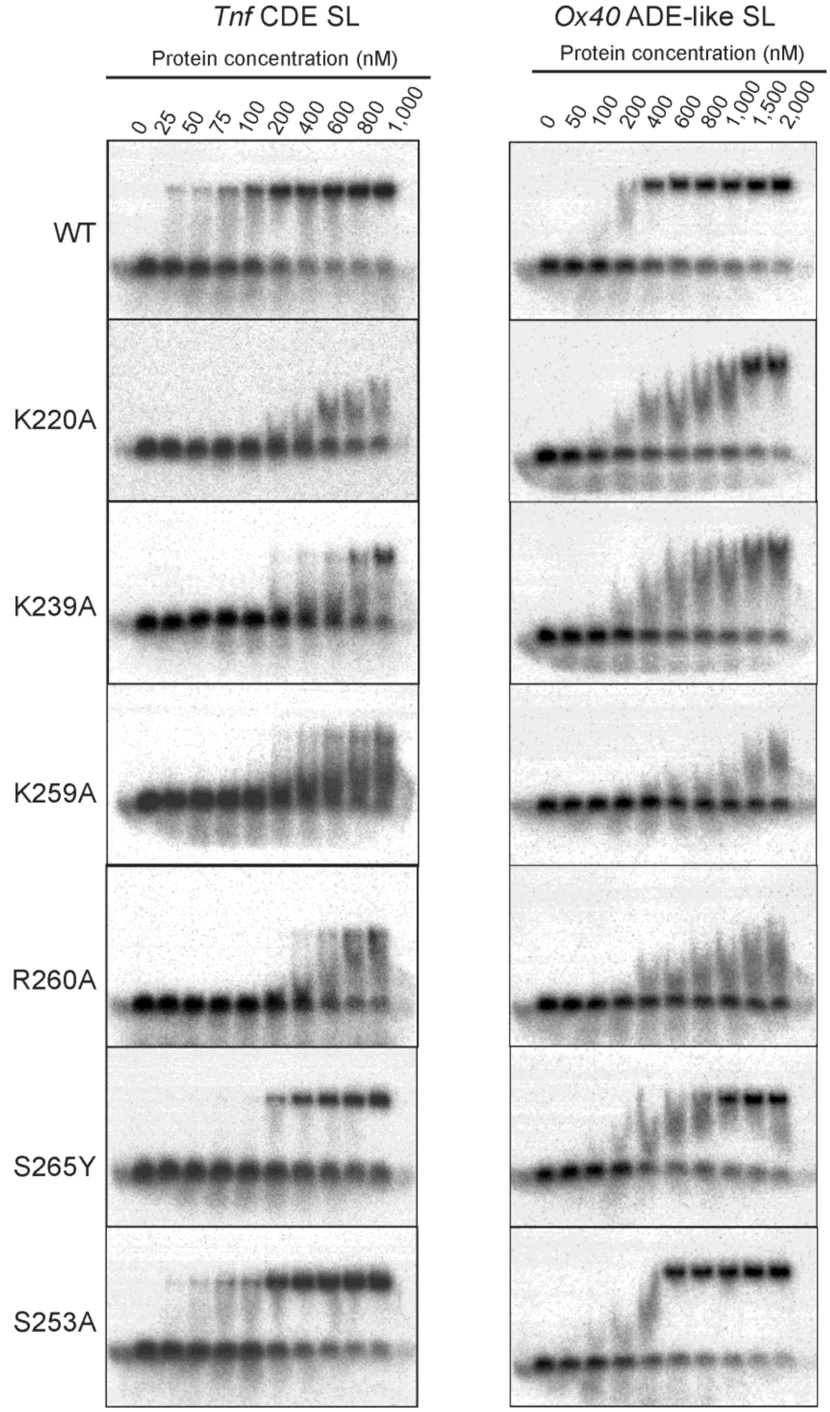

EMSA-based comparison of Roquin-1 ROQ domain mutants and their effect on complex formation with either the previously described *Tnf* CDE SL (left side) or the *Ox40* ADE-like SL RNA motif (right side). The EMSAs with the Y250A mutant are shown in main text **Fig. 5a**.

Supplementary Fig. 5

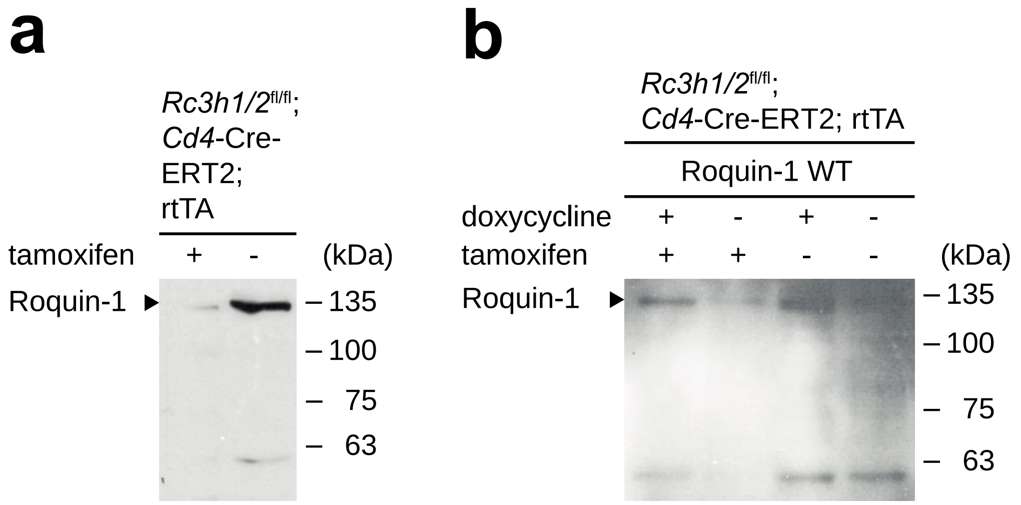

Immunoblot analysis of Roquin expression in T_H1 cells from *Rc3h1/2*$^{fl/fl}$; *Cd4*-Cre-ERT2; rtTA mice. **(a)** Representative Immunoblot analysis of Roquin in lysates of *Rc3h1/2*$^{fl/fl}$; *Cd4*-Cre-ERT2; rtTA T_H1 cells showing tamoxifen-induced deletion of *Rc3h1/2*$^{fl/fl}$. **(b)** Immunoblot analysis of doxycycline-induced reconstitution of Roquin-1 WT expression in *Rc3h1/2*$^{fl/fl}$ expressing (-tamoxifen) or *Rc3h1/2*$^{fl/fl}$ depleted (+ tamoxifen) T cells.

Supplementary Fig. 6

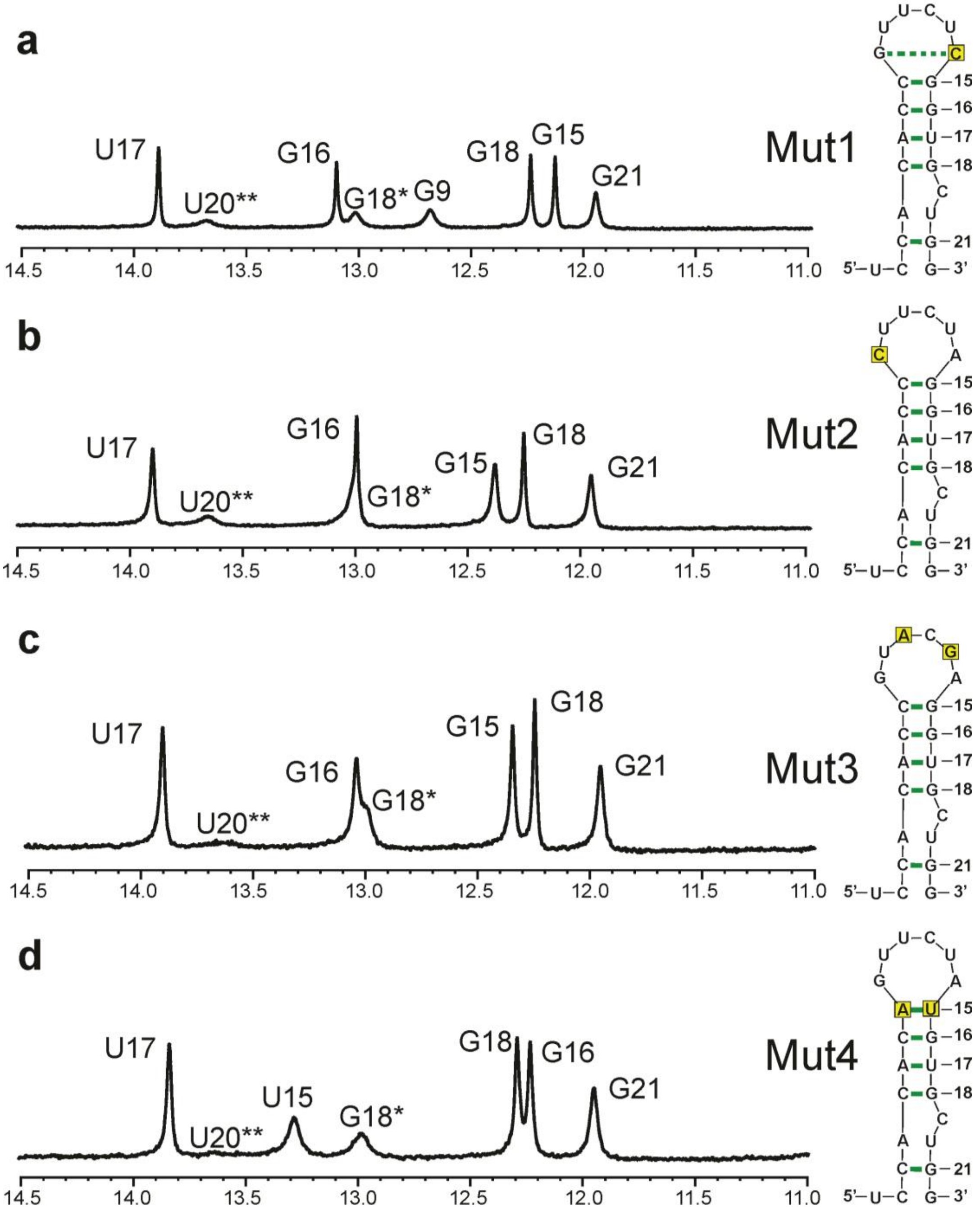

1D-^{1}H-NMR analysis of *Ox40* ADE-like mutant stem-loops. Mutant RNAs are labelled Mut1-Mut4 (panels **a-d**). The spectra show the imino regions of a 1D proton Watergate spectrum and the respective RNA SL is shown next to the spectra. Exchanged nucleotides with respect to the wild-type (see **Fig. 2b**) are boxed in yellow rectangles. Formed H-bonds based on detected imino signals are indicated in green. Asterisks indicate peaks that represent a second conformation (G18*) or transient H-bonding (U20**) close to the bulge formed by C19.

Supplementary Fig. 7

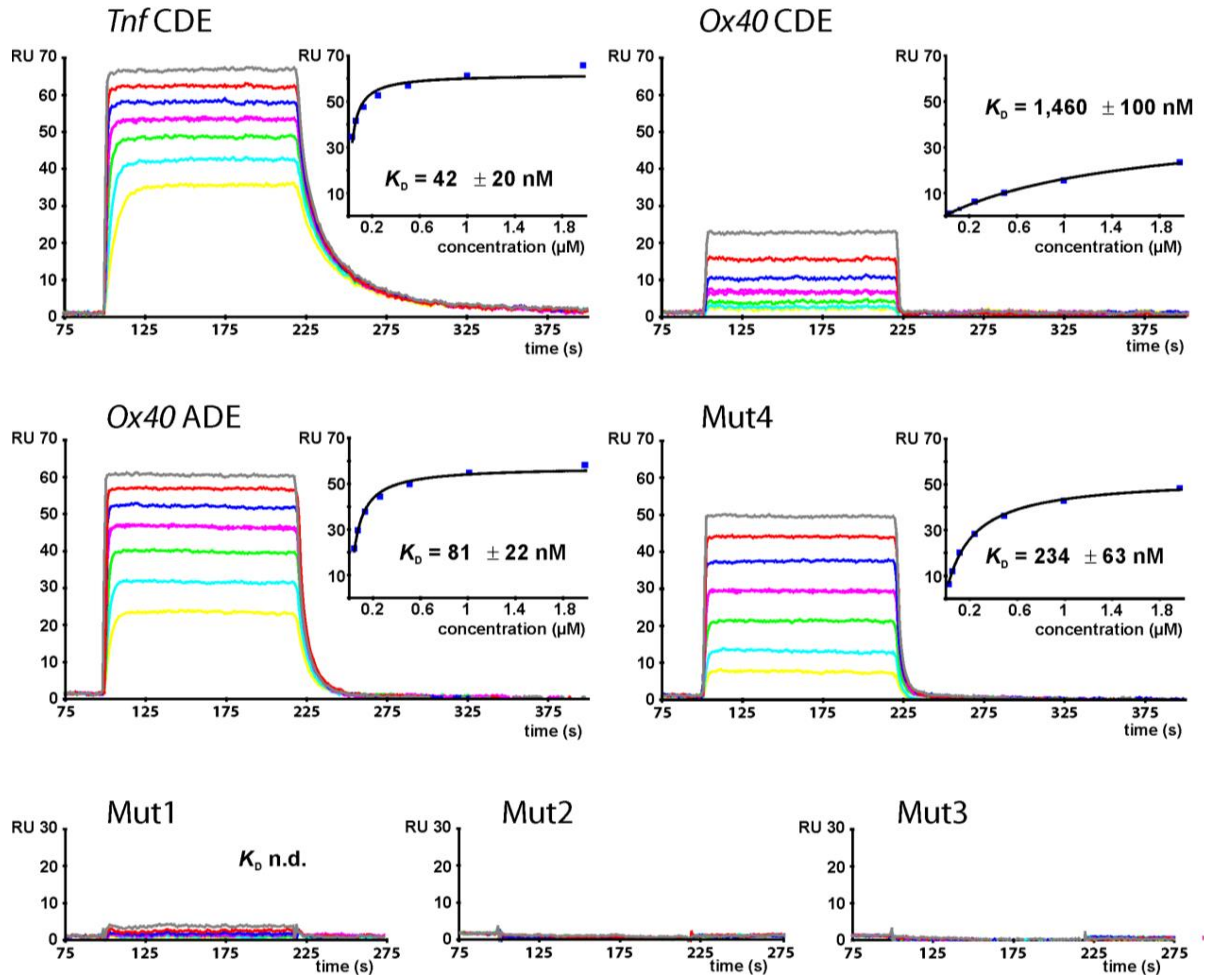

Surface Plasmon Resonance analysis of the Roquin-1 ROQ-domain binding to regulatory RNA motifs from *Tnf* (CDE) and *Ox40* 3'-UTR CDE(-like) and ADE(-like) mRNA elements. Mutant RNAs Mut1-Mut4 refer to the *Ox40* ADE-like motif with nucleotide exchanges as shown in **Supplementary Fig. 6** and **Table 2**. Depicted is a representative sensorgram for each experiment. Insets show binding curves fitted to a one-site-binding model with calculated equilibrium dissociation rates (K_D) and errors as standard deviations from three independent experiments. n.d. is "not determined" because of low total response units (RU) and thus lack of binding. Color coding for individual titration points in as follows: 31.25 nM – yellow, 62.5 nM – cyan, 125 nM – green, 250 nM – magenta, 500 nM – blue, 1 µM – red, 2 µM – gray.

Supplementary Fig. 8

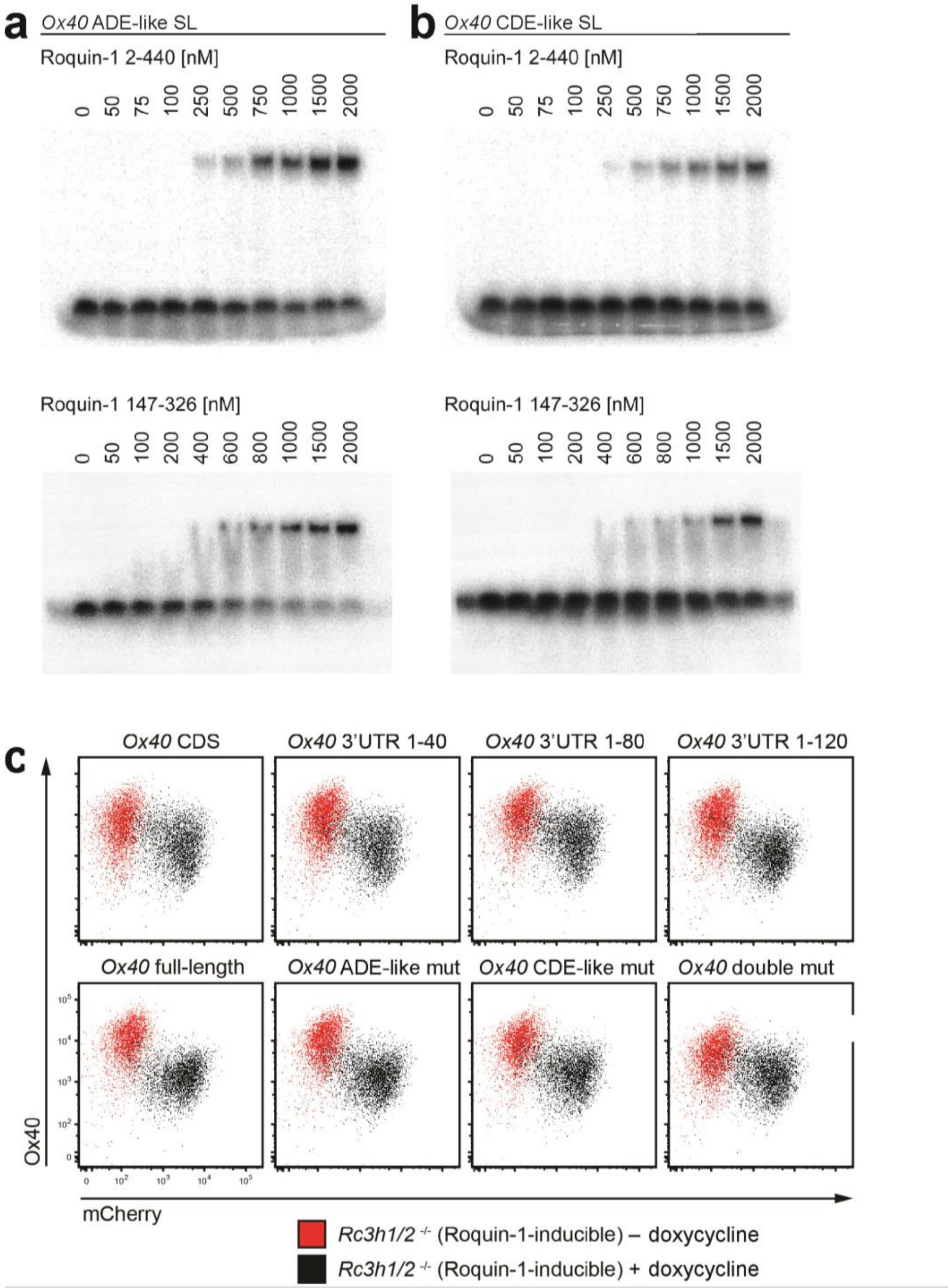

EMSA experiments of Roquin-1 Nterm (2-440, top panels) and ROQ domain (147-326, bottom panels) with the *Ox40* ADE-like (**a**) or the CDE-like SL (**b**). (**c**) Representative dot plots showing expression levels of Ox40 depending on the expression construct (indicated above each plot) and on doxycycline-induced expression of Roquin-1-p2A-mCherry in Rc3h1-2-/- MEFs. The quantification is shown in the bar plot in main text **Fig. 6c**.

Supplementary Notes

Structural features in the ROQ-stem-loop complexes

In addition to the structural features described in the main text, large CSPs are observed for ROQ residues 209 and 210. The NMR signals of these residues are severely line-broadened in the ROQ complex with the *Tnf* tri-loop CDE, presumably due to some conformational dynamics in the binding interface[1]. The fact that the NMR signals of these residues are observed with the SELEX-derived hexa-loop SL RNAs (ADEs) indicates a more rigid and tight interaction at this site.

The imino NMR spectra of the ADE(-like) SLs bound to the ROQ domain (**Fig. 2**) are consistent with the RNA conformation and confirm the base pairs seen in the crystal structures. There are few differences; in particular in the bulge region of the *Ox40* ADE-like RNA. The stem region appears to be dynamic in solution, and this may explain the decreased affinity to the ROQ domain when compared to the *Tnf* CDE SL RNA. A dynamic base-pair could be beneficial for single-nucleotide interactions as seen for the flipped-out U1 in the consensus ADE SL RNA motif (see **Figures 2 and Supplementary Fig. 2f**). This could also explain the presence of a second conformation for the neighboring G2-U19 base-pair within this RNA motif (**Fig. 2a**).

Supplementary References

1. Schlundt A, Heinz GA, Janowski R, Geerlof A, Stehle R, Heissmeyer V*, et al.* Structural basis for RNA recognition in roquin-mediated post-transcriptional gene regulation. *Nat Struct Mol Biol* 2014, **21**(8)**:** 671-678.
